# A human pilot study on positive electrostatic charge effects in solid tumors of the late-stage metastatic patients

**DOI:** 10.3389/fmed.2023.1195026

**Published:** 2023-10-17

**Authors:** Ashkan Zandi, Fatemeh Shojaeian, Fereshteh Abbasvandi, Mohammad Faranoush, Robab Anbiaee, Parisa Hoseinpour, Ali Gilani, Mohammad Saghafi, Afsoon Zandi, Meisam Hoseinyazdi, Zahra Davari, Seyyed Hossein Miraghaie, Mahtab Tayebi, Morteza Sanei Taheri, S. Mehdi Samimi Ardestani, Zahra Sheikhi Mobarakeh, Mohammad Reza Nikshoar, Mohammad Hossein Enjavi, Yasin Kordehlachin, S. M. Sadegh Mousavi-kiasary, Amir Mamdouh, Mohammad Esmaeil Akbari, Masud Yunesian, Mohammad Abdolahad

**Affiliations:** ^1^Nano Electronic Centre of Excellence, Nanobioelectronic Devices Laboratory, Cancer Electronics Research Group, School of Electrical and Computer Engineering, Faculty of Engineering, University of Tehran, Tehran, Iran; ^2^Nano Electronic Centre of Excellence, Nanoelectronics and Thin Film Laboratory, School of Electrical and Computer Engineering, Faculty of Engineering, University of Tehran, Tehran, Iran; ^3^School of Medicine, Shahid Beheshti University of Medical Sciences, Tehran, Iran; ^4^Department of ATMP, Breast Cancer Research Centre, Motamed Cancer Institute, ACECR, Tehran, Iran; ^5^Cancer Research Centre, Shahid Beheshti University of Medical Sciences, Tehran, Iran; ^6^Pediatric Growth and Development Research Centre, Institute of Endocrinology and Metabolism, Iran University of Medical Sciences, Tehran, Iran; ^7^Cardio-Oncology Research Centre, Rajaie Cardiovascular Medical and Research Centre, Iran University of Medical Sciences, Tehran, Iran; ^8^Department of Radiation Oncology, Imam Hossein Hospital, Shahid Beheshti University of Medical Sciences, Tehran, Iran; ^9^SEPAS Pathology Laboratory, Tehran, Iran; ^10^Department of Otolaryngology, Head and Neck Surgery, Taleghani Hospital, Shahid Beheshti University of Medical Sciences, Tehran, Iran; ^11^Medical Imaging Research Centre, Shiraz University of Medical Sciences, Shiraz, Iran; ^12^Department of Radiology, Shohada Hospital, Shahid Beheshti University of Medical Sciences, Tehran, Iran; ^13^Department of Psychiatry, Behavioural Sciences Research Centre, Imam Hossein Hospital, Shahid Beheshti University of Medical Sciences, Tehran, Iran; ^14^Department of Quality of Life, Breast Cancer Research Centre, Motamed Cancer Institute, ACECR, Tehran, Iran; ^15^Department of Gastroenterology Surgery, Taleghani Hospital, Shahid Beheshti University of Medical Sciences, Tehran, Iran; ^16^Department of Environmental Health Engineering, School of Public Health, Tehran University of Medical Sciences, Tehran, Iran; ^17^Department of Research Methodology and Data Analysis, Institute for Environmental Research, Tehran University of Medical Sciences, Tehran, Iran; ^18^Imam-Khomeini Hospital, Tehran University of Medical Sciences, Cancer Institute, Tehran, Iran; ^19^UT&TUMS Cancer Electrotechnique Research Centre, YAS Hospital, Tehran, Iran

**Keywords:** neoplasms, static electricity, neoplasms metastasis, palliative care, therapeutics, clinical study, noninvasive treatment, complementary medicine

## Abstract

**Background:**

Correlative interactions between electrical charges and cancer cells involve important unknown factors in cancer diagnosis and treatment. We previously reported the intrinsic suppressive effects of pure positive electrostatic charges (PEC) on the proliferation and metabolism of invasive cancer cells without any effect on normal cells in cell lines and animal models. The proposed mechanism was the suppression of pro-caspases 3 and 9 with an increase in Bax/Bcl2 ratio in exposed malignant cells and perturbation induced in the KRAS pathway of malignant cells by electrostatic charges due to the phosphate molecule electrostatic charge as the trigger of the pathway. This study aimed to examine PECs as a complementary treatment for patients with different types of solid metastatic tumors, who showed resistance to chemotherapy and radiotherapy.

**Methods:**

In this study, solid metastatic tumors of the end-stage patients (*n* = 41) with various types of cancers were locally exposed to PEC for at least one course of 12 days. The patient’s signs and symptoms, the changes in their tumor size, and serum markers were followed up from 30 days before positive electrostatic charge treating (PECT) until 6 months after the study.

**Results:**

Entirely, 36 patients completed the related follow-ups. Significant reduction in tumor sizes and cancer-associated enzymes as well as improvement in cancer-related signs and symptoms and patients’ lifestyles, without any side effects on other tissues or metabolisms of the body, were observed in more than 80% of the candidates.

**Conclusion:**

PECT induced significant cancer remission in combination with other therapies. Therefore, this non-ionizing radiation would be a beneficial complementary therapy, with no observable side effects of ionizing radiotherapy, such as post-radiation inflammation.

## 1. Introduction

Previous studies reported the role of positive electrostatic charge treating (PECT) in the selective suppressing effect of the proliferation and invasion of malignant tumors with an efficacy of more than 90% on cancerous cell lines and mouse models (1–4). The advantage of PECT in comparison with other electrical field-based cancer therapies, such as electrochemical treatment or irreversible electroporation, is that there is no requirement for current flow into the body (which might be dangerous if it becomes noncontrollable (5, 6)). Hence, PECT is the first safe electric field-based therapeutic method with high potential value and extremely low current (1–4).

Many mechanisms were hypothesized and studied to clarify the reason for such results. In addition to apoptotic pathway activation in malignant cells, which has shown an intrinsic tendency to charge sources and result in loss of tissue attachment (1–3), the role of intrinsic electrostatic charge in one type of phospholipid membrane, which tunes the KRAS and MAPK mitotic pathways in malignant cells was revealed (7). Many parameters such as adhesion, cytoskeletal assemblies, proliferation, and metabolic pathways were assayed on mouse models exposed to positive electrostatic charges (PECs) with specific dose and duration, and the treatment efficacy have been reported with the various parameters compared to radiotherapy (RT) and chemotherapy (3).

In this research, the PECs were examined as a complementary treatment for 41 patients with various types of solid metastatic tumors, who showed resistance to chemotherapy and RTs. Their most vital organs involved were exposed to PECs. Their crucial parameters, such as clinical and radiological manifestations, serological and cancer-associated enzyme values, tumor sizes, and quality of life, were followed. PEC treating (PECT) showed significant targeted remission with no side effects on the normal organs. Its convenience of use with no need for hospitalization, the capability of utilization all day long without any side effects, and the ability to use in every stage of cancer put up PECT as a local complementary therapeutic method, in combination with other treatments, for interfering cancer.

## 2. Materials and methods

### 2.1. Study design and patient selection

Positive electrostatic treating (PECT) study, a non-interventional complementary cancer treatment for patients with advanced cancer, was launched in 2019–2020. Locally advanced or late-stage metastatic cancer patients (stage III or IV), who were not eligible for conventional treatment (including surgery, chemotherapy, or RT), failed with the conventional treatment, or did not consent to perform them due to their side effects, with a performance status of 0–4 (evaluated by the oncologists) were candidates for the study.

Exclusion criteria were as follows: pregnancy, impaired complete blood cell count (CBC) or serum electrolytes, any sign or symptom of infection, cardiac arrhythmia, benign tumors, tumors located at a depth greater than 12 cm beneath the body’s surface, alcohol or substance abuse, and patients without appropriate general condition or a followable solid tumor.

Eventually, 41 patients were admitted to the hospital, and 36 of them continued to meet the inclusion criteria. Patients generally underwent a blood test and imaging investigation 30 and 15 days before the initiation of the therapy, followed by days 0, 6, and 12 of the PECT. They were also followed for any possible side effects and the characteristics of their tumors during the 6 months after PECT. The specialists recommended the patients for the study, and they were admitted to the hospital for a careful and proper investigation during PECT. The team’s specialists and scientists chose the most appropriate region of the body for receiving PECT based on different factors, including the organs’ vitality, applicable patch connection, and the superficiality of the tumor. The most appropriate organs for receiving PECT were the breast and liver (Breast and Liver Cohorts).

All the participants were informed with adequate information about the therapeutic procedure and protocols, their legal rights, services, and all the advantages and disadvantages of the method via a written consent form designed for this study, using FDA and NICE guidelines. Patients had an adequate amount of time for decision-making. All the patients were aware of the results during participation and were informed of the publication of their images and information; the confidentiality of patients’ data was maintained. The Tehran University of Medical Science Ethical Committee (under Ethical code of IR.TUMS.VCR.REC.1397.354 and clinical study registration No. IRCT20190904044697N2) approved the study, and the study methods and protocols were carried out following the guidelines related to the “Managements of Patients with Advanced Metastatic and Late-Stage Cancer.”

### 2.2. Positive electrostatic charge treating

Patients with solid malignant tumors were candidates for PECT based on the mentioned inclusion and exclusion criteria. As previously mentioned, a team of specialists and scientists chose the most appropriate region for receiving PECT based on a variety of aspects, including applicable patch connection, and the superficiality of the tumor, and then the chosen area underwent PECT. One course of PECT was defined as receiving PEC from a metallic patch on the tumor site continuously for 12 days, except for daily routines.

The decision to implement a 12-day course of PECT was based on several factors. Firstly, findings from our prior preclinical research demonstrated the complete eradication of tumors in mouse models following 10 to 12 days of continuous electrostatic voltage application (with no residual tumor bed presence) (3). Secondly, this 12-day duration aligns with conventional cancer treatment cycles such as chemotherapy (14 to 28-day cycles) and radiotherapy (multiple weeks, 5 days a week for 6–7 weeks), allowing for seamless clinical integration. This synchronization offers clinicians flexibility to combine PECT with established treatment regimens, potentially enhancing therapeutic outcomes. Moreover, given the advanced cancer stage of our patient cohort, a 12-day initial treatment course was deemed suitable, with provision for additional PECT courses after a break for patients requiring extended treatment.

We used a flexible patch designed with a sheet metal charged by a DC electrical power system (30kv) to produce accumulated positive charges. To prevent current (charge) leakage, the metal sheet was covered entirely with an electrical isolation biocompatible layer called polydimethylsiloxane (PDMS, Dow Corning SYLGARD 184). The Patches were located on top of the human’s skin on the tumor region, fixed with anti-allergic tape/glue (anti-allergic surgical tape, Micropore Company), and connected to the electrostatic charge generator via a coaxial cable (high voltage DC cable, KDK KAWASAKI-Y 30 kV DC 22AWG). The patients underwent one course (24-h treatment for 12 days), except for daily routines such as eating and bathing. Patients were given total positive charges of 30 kV (480 nC, Supplementary Data Sheet) over 12 days. The patients were under close observation during the treatment. Subsequently, the targeted tumors’ size variations were compared with those outsides of the patch’s area or with themselves before the treatment, if applicable. Other outcomes, such as clinical manifestations, blood tests results, quality of life, and possible side effects, were also assessed. Based on the recorded results, patients were candidates to get another 12 days of PECT (additional course) or not. Supplementary Table 2 shows the detailed characteristics of each patient’s PECT course. Moreover, Supplementary Figures S1–S3 in supplementary information show the simulation of electrostatic field distribution in the human body. Simulations have been conducted over a broad range of applied electrostatic voltages (1–100 kV). Among these, 30 kV is the very first voltage at which we observed a very uniform distribution of the electric field within the body, extending up to a depth of 15 cm from the skin (Supplementary Figures S1, S2). Notably, this depth surpasses the maximum depth of the tumors we have worked with, which is 12 cm. Therefore, in order to mitigate any potential adverse effects of PECT, we have chosen to employ a voltage of 30 kV.

### 2.3. Patient investigation and follow-up

After patients were included in the study, demographical data of the patients, radiological and histopathological features of the tumor, tumors elasticity, clinical manifestations of the patients (e.g., pain, nausea, and wound condition), patients’ tumor markers, and other serum factors were measured 30 and 15 days before the PECT course, followed by days 0, 6, and 12 of the PECT. Their vital signs and further clinical assessment (participants’ signs and symptoms, such as pain, and nausea) were evaluated per 6 and 12 h, respectively. They all underwent blood tests and serial imaging (such as sonography, CT scan, and mammography, based on their underlying diseases, varied for each person) every 6–12 days.

Recent studies demonstrated the application of elastography in investigating different diseases (8–11). For instance, it has been added to the sonography systems to evaluate the elasticity and improve diagnostic accuracy, especially in breast tumor disease (12, 13). Elastography is used with ultrasonography as an adjunctive method to differentiate benign and malignant lesions (12, 14). Malignant lesions are stiffer than benign ones (15) due to their architecture of highly proliferative cancer cells. Hence, we used this feature for further daily evaluation of patients with breast tumors checking for tumor elasticity with a durometer (REX GAUGE CO., US), on top of the tumor site at a specific spot.

Moreover, specialists visited the patients at specific intervals or when new symptoms or signs appeared. Their cardiac, renal, pulmonary, and liver functions have been investigated by blood tests and additional imaging, such as echocardiography, chest x-ray, and CT-angiography. The patients were followed for 6 months after the study regarding the quality of life, tumor characteristics such as size and pathological marker, signs and symptoms, and recurrency. All the evaluated data were reported to the patients’ specialists to decide on the treatment plan.

### 2.4. Histological analysis and IHC

The excised tumor was fixed in 10% formalin, dehydrated in graded alcohol solutions, embedded in Paraplast plus (Oxford Labware, Memphis, TN, USA), and cut into 5 μm thick serial sections. The histological hematoxylin and eosin staining (H&E) method was used for morphological and pathological evaluation of the tumor samples. Each slide was photographed using a camera coupled to the microscope (Olympus-BX51BX51, America, Centre Valley PA) for further qualitative pathological studies at 400X magnification (16).

Immunohistochemical staining: paraffin-embedded tumor sections were deparaffinized with dimethyl benzene after 20 min, and rehydration was performed in graded ethanol concentrations (100, 90, 80, and 70%). Then the immunohistochemical process was done on the sections same as the H&E sections. Briefly, after blocking endogenous peroxidase (3% hydrogen peroxidase), the sections were treated with primary mouse anti-human monoclonal Ki67 and p53 antibodies (1:100), which were diluted in phosphate-buffered saline containing 0.1% Tween-20 (PBST) and 5% bovine serum albumin. After overnight incubation at 4°C, the sections were treated with secondary antibody (1:100), avidin-biotin-peroxidase, and DAB agent. Finally, hematoxylin dye was used for counterstaining, and the sections were visualized by microscopy (Olympus-BX51BX51, America, Centre Valley PA) (16).

### 2.5. Data analysis and statistics

The statistical analysis was performed using SPSS version 25.0 (IBM Corp., Armonk, NY, USA) and GraphPad Prism version 9.0 software. Each patient’s data was presented separately by OriginPro 2018. The before and after PECT values were compared using the paired t-test. The level of significance was set at *p* < 0.05.

## 3. Results and discussion

Based on earlier studies on the effects of the electrostatic field on cancerous cell lines and mouse models, we have hypothesized that the positive electrostatic charge has an intrinsic ability to induce apoptosis in cancer cells in different ways (2, 3). The downregulation of CD31, HIFa-1, and Ki-67 (as glycolytic-based angiogenesis, hypoxia-based metabolic, and proliferative markers, respectively), and upregulation of P21, P16, and P53 (apoptosis-related transcriptomes) in tumor cells exposed to PEC, as well as the suppression of pro-caspases 3 and 9 with an increase in Bax/Bcl2 ratio, were in line with our hypothesis regarding apoptosis induction (1–3).

Previous studies indicated that tumor cells are negatively charged, while the normal cells are neutral or slightly positive (17, 18); hence they are expected to behave differently in electrical field exposure. Therefore, in addition to activation of apoptotic pathways, intrinsic tendency to charge sources and loss of attachment to the tissue were also observed (3). In line with our hypothesis, the role of intrinsic electrostatic charge in one type of phospholipid membrane, which tunes the KRAS and MAPK mitotic pathways in malignant cells, was also revealed in other studies (7). Another research showed that the pure positive electrostatic stimulation induces the deterioration of circulating cancer cells in the blood, while it has no impact on normal cells (4). It assumed that a high-intensity electrostatic field changes the ionic flow and depolarizes the cell surface charge, resulting in an ionic imbalance in invasive, proliferative, and tumorigenic cancer cells and deterioration of their ionic channels (2, 3).

Our previous research, while primarily centered on breast cancer, extends to a wide array of cell lines, each with its unique characteristics. These encompass skin (SK-MEL-37), ovary (OVCAR-3), cervix (HeLa), glioblastoma (U-87 MG), stomach (KATO III), prostate (PC-3), colon (SW 48), and kidney (ACHN) cells (19). An intriguing trend emerged - the more malignant the cell line is, the more pronounced the response to our electrostatic therapy. This observation was particularly evident when comparing different breast cancer cell categories, where we noted significantly more robust effects on triple-negative breast cancer cells compared to their triple-positive counterparts (2, 3). These varying responses to the same applied voltage and charge among different cell lines can be attributed to several factors. Firstly, more malignant cells often carry a higher negative charge due to their elevated metabolic rates and acidic environments, making them more susceptible to a positively charged field (20, 21). This heightened electrostatic interaction allows our therapy to exert a more profound influence on these cells compared to those with lower negative charges. Secondly, highly malignant cells typically exhibit increased metabolic activity, leading to more complex cellular signaling and greater electrostatic and mechanical interactions within the cell (22, 23). Consequently, an applied electrostatic voltage has the potential to disrupt this intricate signaling network to a greater extent than in less malignant or normal cells. Understanding these nuances enables us to customize electrostatic therapies for specific malignancies effectively. We can adapt treatment parameters, including voltage and charge levels, depending on the cancer type, size, and location, thus paving the way for precise and tailored therapeutic approaches in the future.

These shreds of evidence favor the PEC effects on malignant cells and their underlying cause. Consequently, as the PECT showed not only an impressive effect on cancer suppression but also no side effects on other organs of the mouse models (3), in the next step, we administered PECT to candidate patients with late-stage metastatic cancer, under approved care protocols.

### 3.1. Localized PECT in cohort of metastatic breast cancer patients with multiple tumors in breast and related lymph nodes

**Figure 1:**
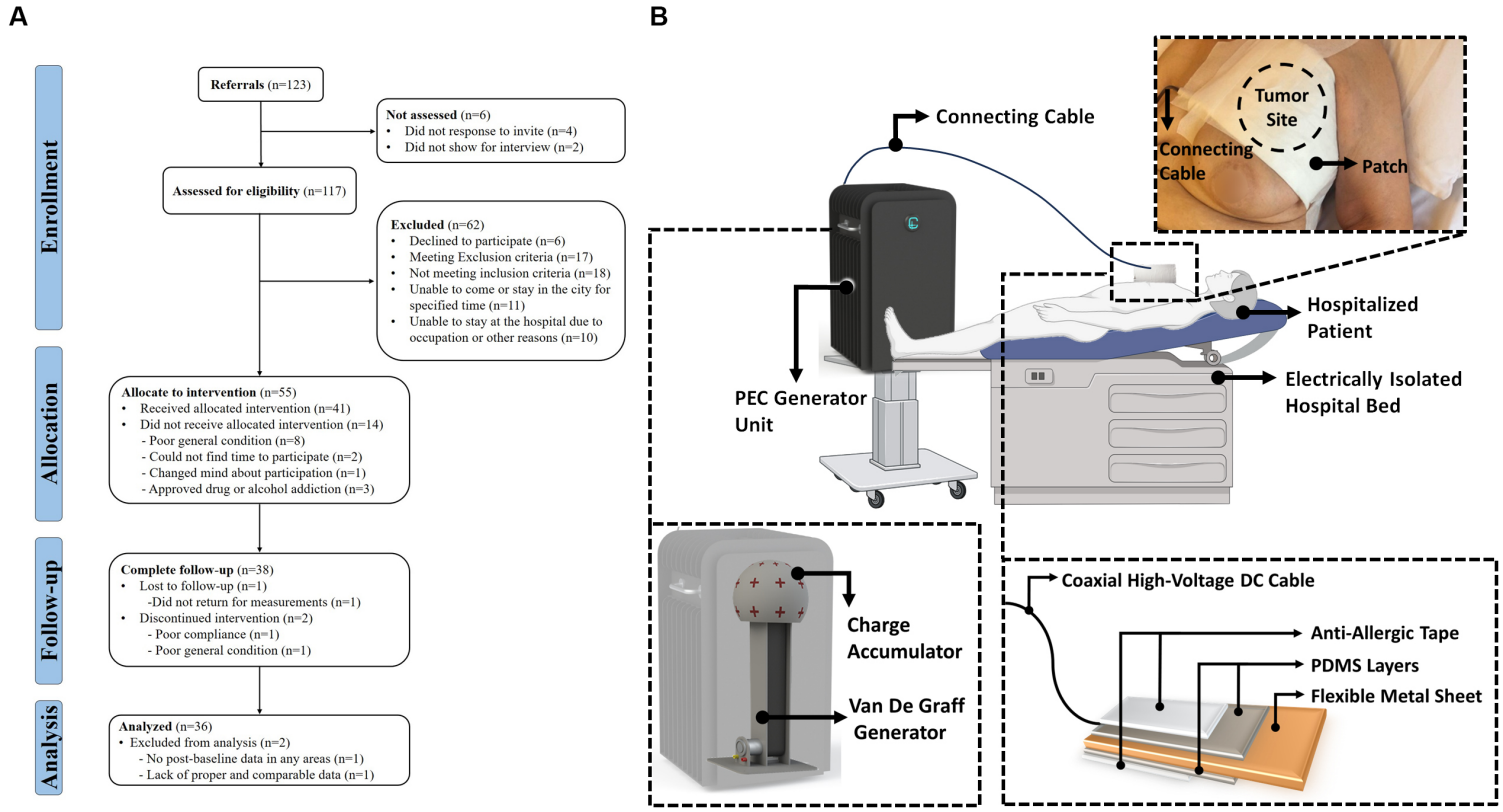
**(A)** TREND flow diagram of the positive electrostatic charge complementary therapy, human study. **(B)** Schematic representation of the PECT setup. A hospitalized patient is depicted alongside the PEC generator unit. A patch is applied to the patient’s skin over the tumor site and is connected to the generator unit *via* a connection cable. The detailed diagram zooms in on the patch, revealing its multiple layers. These layers consist of anti-allergic surgical tape and PDMS both serve to protect and insulate a flexible metallic sheet contained within the patch. This metallic sheet plays a crucial role in accumulating PEC directly over the tumor site as part of the therapeutic process.

The human study of PECT was a pilot clinical study on the PEC effects on patients with late-stage malignant tumors. Figure 1A depicts the TREND flow diagram, which elaborates the four stages of the survey (enrolment, intervention allocation, follow-up, and analysis). The TREND diagram explicitly shows the number of participants, exclusions, and inclusions during the mentioned four stages. The schematic in Figure 1B illustrates the application of PECT to a hospitalized patient.

**Figure 2:**
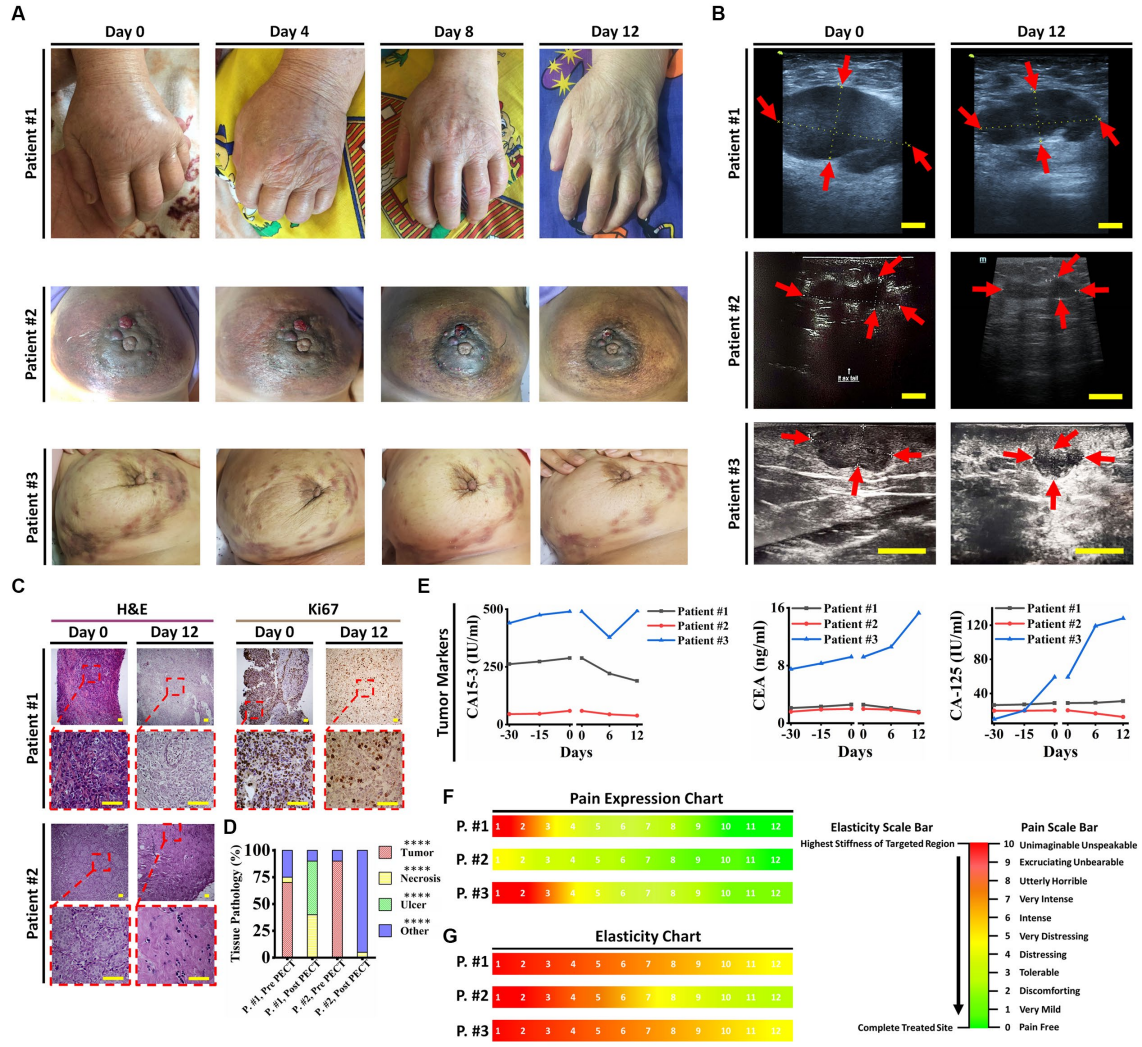
Patient ID#1 was an 83-year-old woman with the diagnosis of invasive ductal carcinoma metastasized to axillary lymph nodes, left supraclavicular lymph nodes and left iliac crest (traced in PET scan). Pathological evaluation of the core needle biopsy on the tumor showed a dense distribution of cancer cells with high N/C, ER: 10%, PR: 10%, HER-2/neu: negative, Ki67: 90%. She had severe pain and edema in her left limb, with muscle and nerve atrophy due to immobilization of her hand, in addition to anorexia, general weakness, and sleeping difficulties, which affected her quality of life. The patient’s geriatric conditions prevented her from receiving interventional surgery or chemo/radiotherapies; she just received a limited dose of Letrozole to suppress further tumoral angiogenesis. Her disease progression has made her incapable of handling daily routines. Due to her advanced disease and poor prognosis, the oncologists provided the patient with only supportive and palliative care. Her estimated life expectancy was less than 2 months. Hence, she was a candidate for PECT on her left (upper outer quadrant, UOQ) and metastatic axillary lymph nodes (ALNs). PECT induced a drastic reduction in her tumor sizes, edema, pain, and CA15-3 antigen blood level. Moreover, the PECT increased the tumor elasticity as a crucial factor in favor of response to this complementary treatment. As PECT has an electrical nature and might interact with the ionic species of the body, the crucial ionic states of the blood (serum electrolyte) were followed. It did not induce any perturbation in the levels of serum electrolytes of the patient. Furthermore, no other symptoms in favor of side effects were observed. After PECT, the lymph nodes became painless (without painkillers) with no pain recurrence, and the circumference of the left hand (exposed hand) was significantly decreased. Apart from tumor reduced sizes and improved pathological parameters in favor of treatment, significant improvement in the patient’s lifestyle and reduced tumor adhesion persuaded the team’s surgeons to suggest core needle biopsy (CNB) for the patient to evaluate the exposed tumor function and feasibility of the surgery. Pathological results showed extensive necrosis (about 50%) in breast tumors and axillary lymph nodes and reduced Ki67 expression. PECT changed the patient’s therapeutic plan from palliative care to a new one that included surgery, followed by chemotherapy. In the following, patient underwent post-surgical chemotherapy, and she was alive (after 8 months) with a normal lifestyle. In conclusion, an end-stage cancer patient survived and returned to a stable lifestyle with the assistance of PECT as an effective adjuvant therapy in combination with surgery and chemotherapy. Patient ID#2 was a 40-year-old woman with a history of locally advanced triple positive (ER +, PR +, HER-2/neu 3+) tumors in her left breast. The patient had undergone surgery followed by post-surgical chemotherapy. One year later, recurrence occurred at her cervical lymph nodes and the surgical site, while its pathological characteristics were transformed to triple-negative. The patient suffered from severe areola and ALN involvement. She had not benefited from previous chemotherapies. Patient ID#3 was a 58-year-old woman with a two-year history of multiple tumors in her right breast with ALN, chest, ribs, and lung metastasis, declined to do surgery and chemotherapy due to their side effects. One year of receiving herbal substances resulted in no suppression of her disease progression. **(A)** Optical images of patients 1–3 (on days 0, 4, 8, and 12 of PECT) demonstrate drastic changes in the PECT targeted organs. **(B)** Ultrasonography images of the patients 1–3 at the beginning and after PECT. Arrows indicate targeted lesions before and after PECT. Scale bars, 1 cm. **(C)** H&E and Ki67 staining of patients ID#1 & ID#2 biopsied tumors before and after PECT. Scale bars, 20 μm. **(D)** Pathological tissue components of the patients’ ID#1 & ID#2 biopsied tumors before and after PECT. The mean of 10 different slides determined the percentage of each component from one biopsied tumor (****p < 0.0001, *p > 0.05, Paired *T*-test). **(E)** Cancer-associated antigens alterations of patients 1–3 from 30 days before PECT and during PECT. **(F,G)** Pain expression and elasticity chart of the patients 1–3, respectively. The reference intervals of serum biomarkers and blood biochemistry factors are presented in Supplementary Table 3.

Our first cohort of breast cancer patients consisted of three female candidates suffering from progressed breast cancer, manifested as numerous breast solid tumors and conventional therapeutic failure, that PECT was administered to. In brief, they all had life-threatening complications, one of which had severe lymphedema in her left upper limb (patient ID#1) (Figure 2A), and the other had multiple metastatic lesions in her lungs, ribs, and chest wall (patient ID#3). Patient ID#3 could benefit from receiving paclitaxel according to her condition. Still, she declined taking paclitaxel due to her history of severe systematic post-chemotherapy side effects, such as gastrointestinal and respiratory complaints (Supplementary Table 1). Patient ID#2 had undergone a partial mastectomy (breast conserving surgery), but she showed resistance to post-surgical therapies, and her cancer recurred after 6 months. Hence, these three patients underwent PECT on their targeted breasts. Supplementary Table 1 lists the participants’ demographic information, and all the changes are discussed thoroughly in Table 1.

**Table 1:** Patients detailed data (clinical and radiological manifestation, serum marker, and cancer antigen alterations) before and after PECT.

Patient ID	PECT site	Target lesion									Clinical manifestation changes
		Clinic (measured circumference)	Clinic (% of changes)		Radiology	Radiology (% of changes)		Serum markers	Serum marker (% of changes)		
			30 days before PECT to Day 0	Day 0 to After PECT		30 days before PECT to Day 0	Day 0 to After PECT		30 days before PECT to Day 0	Day 0 to After PECT	
8	Both Lung Lobes	Not measurable			Most Prominent Lung Nodule	Increasing in size and number of lung nodules	Completely vanish	ESR	55%	−86%	-No clinical changes
14	Left Breast	Not measurable			Left breast 7 O’clock mass	128%	−57%	ESR	100%	−57%	-Significant reduction in pain and edema of the left breast -Improvement of anorexia, general weakness, and sleeping condition
					Left breast Lower Inner Quadrant mass	57%	−54%	LDH	38%	−39%	
					Left breast Upper Outer Quadrant mass	47%	−34%				
13	Epigastric Wall	Epigastric wall mass	0%	−10%	Epigastric wall mass	34%	−26%	CA125	7%	−17%	-Healing of hysterectomy wound -Significant reduction of ki67 from 30 to 3 -Significant reduction in pain of epigastric wall mass -Improvement of anorexia, general weakness, and sleeping condition
5	Liver	Abdominal circumference	5%	−6%	Left liver lobe mass1	62.5%	−100%	PSA	35%	−19%	-Healing of the abdominal pain -Improvement of defecation condition -Reduction in abdominal circumference (amount of ascites) -Improvement of anorexia, general weakness, and sleeping condition
					Left liver lobe mass2	17%	−100%	AST	239%	−40%	
								ALT	30%	25%	
					Caudate liver lobe mass	52%	−29%	ALP	206%	10%	
								y-GT	46%	10%	
1	Left Breast and left axillary	Left wrist	11%	−11%	Left breast 12 O’clock mass	33%	−52%	CA125	8%	7%	-Healing of the breast and upper limb pain -Significant reduction of left upper limb and breast edema -Decreasing adhesion of tumor and ability to do the mastectomy -Improvement of anorexia, general weakness, and sleeping condition
		Left elbow	30%	−13%							
		Left arm	19%	−17%	Left breast Axillary mass1	6%	−23%	CA15-3	9%	−35%	
		Right wrist	1%	−5%							
		Right elbow	3%	−5%	Left breast Axillary mass2	7%	−49%	CEA	22%	−38%	
		Right arm	2%	−4%	Left breast Upper Outer Quadrant mass	28%	−35%				
9	The wound of right upper back	Wound area on the skin of the right upper back	100%	~ − 90%	No mass			CA125	44%	−31%	-Significant reduction in pain of ulcers -Ability to sleep supine (on the wound) -Healing of the wound and decrease in wound area -Improvement of anorexia, general weakness, and sleeping condition
								CA15-3	17%	−37%	
								CEA	57%	−6%	
4	Liver	Abdominal circumference	2%	−5%	Segment 7 liver mass	83%	−25%	CA125	46%	−11%	-Significant reduction in abdominal pain -Reduction in abdominal circumference (amount of ascites) -Improvement of anorexia, general weakness, and sleeping condition
					Segment 7 liver mass	13%	−3%	CA15-3	95%	−8%	
								CEA	492%	−6%	
26	Liver	Not measurable			Right liver lobe mass	36%	−32%	CA125	231%	104%	-Abdominal and lower back pain reduction -Improvement of anorexia, general weakness and sleeping condition
								CA15-3	87%	32%	
								CEA	244%	32%	
								AST	141%	37%	
					Left liver lobe mass	383%	−48%	ALT	75%	22%	
								ALP	85%	16%	
								y-GT	120%	51%	
3	Right Breast	Not measurable			Right Retro areolar mass	10%	−78%	CA125	492%	116%	-Right breast pain reduction -Right breast stiffness and edema reduction -Decreasing in size and number of breast subcutaneous nodules and masses -Improvement of anorexia, general weakness, and sleeping condition
					Right breast 9 O’clock mass	6%	−68%	CA15-3	11%	0.6%	
					Right breast 10 O’clock mass	5%	−20%	CEA	22%	66%	
					Right breast 9-10 mm far zone mass	7%	−66%				
27	Liver	Not measurable			Left liver lobe mass	53%	−48%	CA125	103%	46%	-Capable to walk -Abdominal pain reduction -Improvement of anorexia, general weakness, and sleeping condition
					Right liver lobe mass	18%	−15%	CA15-3	39%	−75%	
					LAP posterior IVC mass	33%	−45%	CEA	0%	33%	
					LAP portahepatis 1 mass	27%	−25%	AST	52%	34%	
					LAP portahepatis 2 mass	21%	−6%	ALT	46%	−41%	
					LAP portahepatis 3 mass	23%	−18%	ALP	39%	36%	
								y-GT	148%	82%	
28	Wound of Right Breast (site of mastectomy)	No mass			No mass			CA125	7%	−14%	-Complete healing of the breast pain -Significant healing of the right mastectomy site wound
								CA15 − 3	2%	−3%	
								CEA	0%	−19%	
								ESR	46%	−50%	
								CRP	75%	> −43%	
20	Neck	Not measurable			Neck lymphadenopathy	10%	−16%	Thyroglobulin	10%	−22%	-Improvement of hoarseness -Improvement of swallowing condition
7	Neck and lung	Not measurable			Neck lymphadenopathy	190%	−77%	AntiThyroglobulin	39%	−15%	-Decreasing neck mass size -Improvement of hoarseness -Improvement of swallowing condition
2	Left Breast	Not measurable			Left Breast Axillary tail mass	50%	−48%	CA125	25%	−39%	Stiffness, edema, pain, and size of left breast reduction
					Left Breast Lateral part mass	8%	−46%				
					Left Breast UOQ mass	117%	−63%	CA15-3	31%	−35%	
					Left Breast UIQ mass	49%	−86%	CEA	2%	−25%	
					Left Breast 6 O’clock mass	25%	62%				
6	Liver	Abdominal circumference	7%	−5%	Spleen Volume	5%	−21%	CA19-9	16%	−24%	-Volume reduction of the splenomegaly -Improvement of anorexia, general weakness, and sleeping condition
					Gallbladder Size	15%	−25%	CEA	46%	−26%	
					Most prominent liver Mass	10%	−3%	AST	48%	−18%	
					Segment 8 liver mass	Because of the liver heterogeneity tumor was not obvious	−25%	ALT	40%	−9%	
					Right liver lobe mass	Because of the liver heterogeneity tumor was not obvious	−100%	ALP	29%	−23%	
								y-GT	9%	−13%	
22	Skin of right breast	No mass			No mass			CA125	31%	19%	-Breast pain reduction -Skin erythema reduction
								CA15-3	9%	7%	
								CEA	17%	40%	
12	Epigastric region	mass circumference in physical examination	25%	−30%	Epigastric mass	66%	−100%	CA19-9	30%	−34%	-Epigastric pain and stiffness of epigastric area reduction -Improvement of anorexia, general weakness, and sleeping condition
								CEA	91%	−39%	
40	Liver	Not measurable			Segment 5 liver mass	58%	0%	CA19-9	21%	−7%	-Abdominal pain healing -Improvement of anorexia, general weakness, sleeping condition
					Segment 8 liver mass	166%	0%	CEA	22%	2%	
					Left liver lobe	62%	−21%	AST	32%	−25%	
								ALT	0%	−20%	
					Right liver lobe mass	55%	29%	ALP	145%	−59%	
								y-GT	59%	1%	
29	Periumbilical	Not measurable			Abdominal right lower quadrant mass	104%	−27%	CA125	58%	41%	-Abdominal pain reduction -Improvement of anorexia, general weakness, and sleeping condition
								CEA	22%	9%	
					Right para umbilical mass	37%	−20%	a-FP	16%	14%	
								HCG	13%	−20%	
24	Periumbilical Region	Abdominal circumference	8%	−1%	Liver mass	28%	25%	CEA	92%	6%	-Capable to walk -Abdominal pain reduction -Improvement of anorexia, general weakness, and sleeping condition
					Spleen mass	98%	40%	CA19-9	194%	13%	
					Periumbilical mass	54%	−1%	AST	92%	111%	
								ALT	62%	53%	
					Pelvic Cavity mass	16%	−9%	ALP	27%	−20%	
								y-GT	35%	−6%	
24	Liver	Abdominal circumference	−1%	−3%	Liver mass	25%	−9%	CEA	6%	6%	-Capable to walk -Abdominal pain reduction -Improvement of anorexia, general weakness, and sleeping condition
					Spleen mass	40%	−7%	CA19-9	13%	5%	
					Periumbilical mass	−1%	6%	AST	111%	33%	
								ALT	53%	40%	
					Pelvic Cavity mass	−9%	30%	ALP	−20%	−11%	
								y-GT	−6%	−4%	
16	Lungs	Not measurable			Lung nodules	Increasing	No significant change	ESR	142%	61%	-Improvement of cough -Improvement of dyspnea from FCIV to FCII -Improvement of anorexia, general weakness, and sleeping condition
								CRP	328%	47%	
33	Left Flank	Not measurable			Excluded from the trial			CA125	50%	36%	-Complete healing of flank pain -Improvement of anorexia, general weakness, and sleeping condition
37	Liver	Not measurable			Segment 2 liver mass	100%	−78%	CA125	28%	−67%	-Capable to walk -Improvement of anorexia, general weakness, and sleeping condition
								CA19-9	30%	−31%	
								AST	12%	6%	
								ALT	20%	−17%	
								ALP	15%	−20%	
								y-GT	40%	−36%	
								ESR	49%	−81%	
								CRP	24%	−83%	
11	Left and Right axilla	Left wrist	20%	−37%	Excluded from the trial			Excluded from the trial			-Significant reduction in upper limbs pain -Significant reduction in upper limbs edema -Improvement of anorexia, general weakness, sleeping condition
		Left elbow	24%	−18%							
		Left arm	18%	−17%							
		Right wrist	10%	−18%							
		Right elbow	17%	−20%							
		Right arm	12%	−14%							
31	Right groin and pubic area	Not measurable			Pelvic cavity mass	Increasing in size and developing new masses on the skin of the head, chest, etc.	stable	ESR	48%	1%	-Right groin pain reduction -Healing of the lower limbs paresthesia -Improvement of anorexia, general weakness, and sleeping condition
								CRP	75%	46%	
41	Left breast	Not measurable			Left breast masses	Increasing in size and number	stable	CA125	20%	−10%	-Reduction in left breast pain and size -Improvement of anorexia, general weakness, and sleeping condition
								CA15-3	67%	−12%	
								CEA	85%	42%	
19	Left upper back	left upper back tumor	90%	−49%	Just clinically measured			Excluded from the trial			-Capable to walk -Healing the pain of the upper back tumor -Decrease the size of the upper back tumor -Ability to sleep in the supine position on the tumor -Improvement of anorexia, general weakness, and sleeping condition
25	Left breast and left upper back	No mass			No mass			Excluded from the trial			-Capable to walk -Significant reduction in pain of ulcers -Ability to sleep supine (on the wound) -Healing of the wound and decrease in wound area -Improvement of anorexia, general weakness, and sleeping condition
10	Frontal mass	Frontal mass	242%	−86%	Just clinically measured			CA125	23%	−39%	-Decreasing frontal mass size -Improvement of anorexia, general weakness, and sleeping condition
18	Left breast mass	Left breast mass	21%	−26%	Just clinically measured			CA125	43%	−11%	-Reduction in the left breast and breast mass pain -Reduction in size and edema of the left breast -Improvement of anorexia, general weakness, and sleeping condition
								CA15-3	26%	−29%	
								CEA	4%	−5%	
35	Liver	Not measurable			Segment 4a liver mass	10%	−30%	AST	10%	5%	-Improvement of anorexia, general weakness, and sleeping condition
					segment 4b liver mass	25%	−26%	ALT	15%	13%	
					Segment 5 liver mass	34%	−57%	ALP	12%	0%	
					segment 6a liver mass	60%	−77%	y-GT	100%	−4%	
					segment 6b liver mass	10%	−14%	CA19-9	51%	21%	
					Segment 8 liver mass	15%	−13%	CEA	110%	4%	
34	Left breast wound	No mass			No mass			CA125	85%	55%	-Reduction in discharge, bleeding, and area of left breast wound -Improvement of anorexia, general weakness, and sleeping condition
								CA15-3	70%	25%	
								CEA	67%	32%	
								ESR	40%	−46%	
								CRP	35%	−19%	
36	Liver	Not measurable			Left lobe liver mass	37.50%	−10%	AST	97%	−43%	-Reduction in pain and tenderness of right upper quadrant -Improvement of anorexia, general weakness, and sleeping condition
								ALT	107%	−43%	
								ALP	144%	−31%	
								y-GT	57%	−29%	
								CA19-9	116%	21%	
								CEA	6%	59%	
17	Lungs	Not measurable			Lung nodules	stable	stable	ESR	50%	31%	-No clinical changes
								CRP	50%	778%	
39	Right breast mastectomy site	Not measurable			Just blood exam has been done			CA125	70%	49%	-Reduction in pain of right breast -Improvement of anorexia, general weakness, and sleeping condition
								CA15-3	65%	33%	
								CEA	10%	0%	
30	Right eyeball	Just microscopic investigation has been done			Just microscopic investigation has been done			ESR	40%	−55%	-Reduction of angiogenesis and abnormal cells in cancerous site -Improvement of anorexia, general weakness, and sleeping condition
								CRP	0%	0%	
15	Excluded from the study										
32	Excluded from the study										
23	Excluded from the study										
21	Excluded from the study										
38	Excluded from the study										

Red color: The primary and secondary values are out of the normal range.

Blue color: The primary and secondary values are in the normal range.

Green color: The primary value is out of the normal range, and the second value is normal.

Orange color: The primary value is in the normal range, and the second value is out of the normal range.

**Figure 3:**
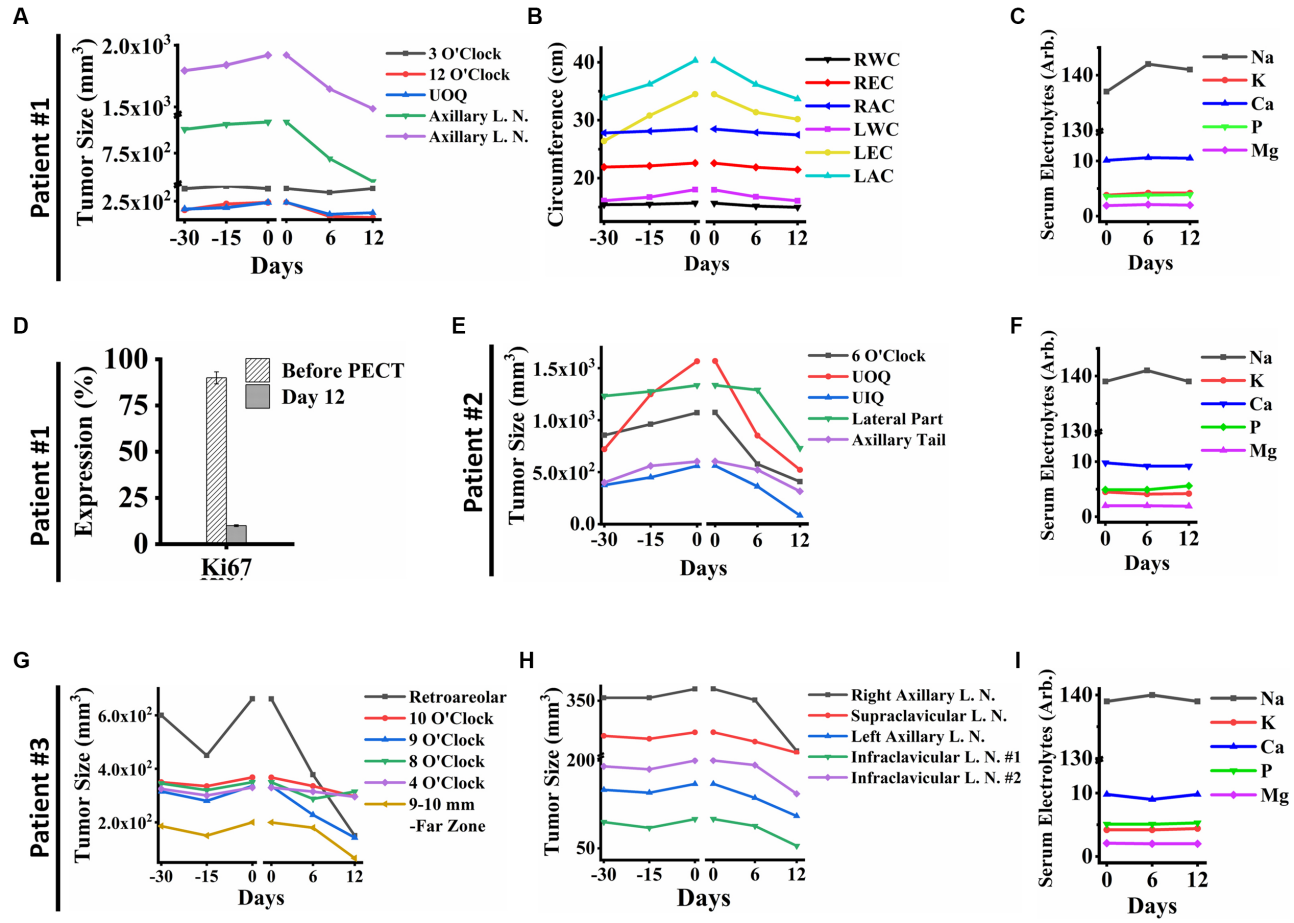
**(A)** Breast tumor size graph of patient ID#1. It showed a significant reduction in the tumors’ size in the PECT site, while PECT did not affect the 3 O’clock mass as a benign tumor. **(B)** The clinical parameters measured in patient ID#1: Right Wrist Circumference (RWC), Right Elbow Circumference (REC), Right Arm Circumference (RAC), Left Wrist Circumference (LWC), Left Elbow Circumference (LEC), and Left Arm Circumference (LAC). They improved with PECT. **(C)** Serum electrolytes of patient ID#1 showed no significant changes during PECT. **(D)** Ki67 expression showed a substantial reduction of proliferation factor in the exposed site before and after the PECT. **(E)** Breast Tumors sizes of patient ID#2. The breast tumors showed a significant size reduction. **(F)** Serum electrolytes of patient ID#2 during PECT. **(G)** Breast tumor sizes of patient ID#3 were demonstrated from 30 days before PECT and during PECT. Tumor in the far zone of the breast vanished completely, and the other ones showed size reduction. **(H)** Lymph node sizes of patient ID#3. The sizes decreased significantly during PECT. Although, they all had shown an upward trend before PECT. **(I)** Serum electrolytes of patient ID#3 during PECT showed no significant changes. The reference intervals of serum biomarkers and blood biochemistry factors are presented in Supplementary Table 3.

In patient ID#1, we observed a rapid improvement in the patient’s general condition, a remarkable reduction in lymphedema and her limb size accordingly (Figure 2A), a significant decrease in her breast tumor size (Figures 2B, 3A–C), a drastic reduction in her pain, emergence of visible gaps between formerly conglomerated axillary lymph node (ALN) tumors, and significant improvement in her quality of life after PECT. Figure 3D shows a ki-67 IHC of the tumor with considerable reduction after PECT, as a proliferative factor. The post-treatment expression of caspase-3 in the patient has been depicted in Supplementary Data Sheet. Notably, the patient’s benign tumor (BIRADS3 at 3 O’clock) did not exhibit any PECT induced size reduction, which is in corroboration with our findings of the selective effect of PECT on just malignant cells (1–3). Supplementary Figure S4 in supplementary data depicts the serial sonography imaging of patient ID#1 tumors.

After applying PECT on patient ID#2, a reduction in her tumor size (Figures 2B, 3E,F), as well as an increase in tumor elasticity (Figure 2A), with no perturbation on serum electrolytes (Figures 3E,F) were observed. The H&E of the tumor biopsies showed extensive necrotic regions in favor of tumor destruction (Figures 2C,D). There was no increase in cancer-associated antigen (Figure 2C).

Lastly, patient ID#3 showed not only a notable reduction in her breast tumor size after PECT (480 nC, 30 kV) but also a meaningful decrease in the size of metastatic lymph nodes (Figures 3G,H,I). Post-PECT reduction of pain and increase in tumor elasticity (Figures 2F,G) were also observed in this patient, with no perturbation on serum electrolytes levels (Figures 3G,H,I). Although we did not observe meaningful changes in the levels of cancer-associated enzymes of the blood after PECT, its increasing slope has been reduced (Figure 2E). The patients’ serum electrolytes levels before and after PECT have not changed significantly (Supplementary Figure S5).

Remarkably, the potential generated by such accumulated pure PECs make severe imbalance in metabolic functions of malignant cells with high mitotic rate and glycolysis energy production pathways. These clinical observations are directly correlated with our previous reports about the role of electrostatic charges in perturbing NRAS and KRAS pathways of malignant cells (3, 7, 24) and/or disassembling the actins and microtubules (the two important electrically active components of the cytoskeleton) to prevent cancer mitosis. The promising results of applying PECT on the breasts of these three patients made us use PECT on ten breast cancer patients, which all showed favorable changes in their disease condition (Table 1 presented the detailed changes in each patient).

Biomarkers and cancer-associated enzymes play pivotal roles in various aspects of breast cancer treatment. Initially, they aid in the diagnosis by helping identify specific breast cancer subtypes and commonly assessed to determine the most appropriate treatment approach. Furthermore, throughout the treatment, these markers serve as critical indicators of treatment response and disease progression (25–27). Regular monitoring of cancer antigens, like CA15-3, in breast cancer patients can help healthcare providers assess the effectiveness of therapies, detect potential relapses, and make timely adjustments to the treatment plan. In essence, biomarkers and cancer antigens provide valuable information that guides clinicians in tailoring treatment strategies to maximize their effectiveness for each breast cancer patient, particularly in precision medicine (28, 29). Specifically speaking, serum markers, including CEA, CA19-9, CA-125, CA15-3, and tissue polypeptide-specific antigen offer diagnostic utility in the context of metastatic breast cancer (MBC), and their diagnostic value varies depending on the specific combinations utilized. In a related study, for diagnosis of MBC, CEA showed the highest sensitivity and CA-125 had the highest specificity when using single marker; in addition, when comparing patients with and without liver metastasis, they found significant differences in positive rate of CA-125 (30). Consistently, in another study, CEA and CA-125 have demonstrated advantages when used in conjunction with CA15-3 for MBC diagnosis and prognosis, emphasizing the importance of considering all three markers in the evaluation of MBC cases (31). Interestingly, panels featuring two markers are found to be more suitable for predicting distant metastasis in breast cancer when compared to panels incorporating three or four markers, while maintaining similar levels of sensitivity and AUC (32). Taken altogether, tumor markers demonstrate their predictive potential for evaluating distant metastasis in breast cancer. The combination of these markers proves especially valuable in augmenting sensitivity compared to relying on a solitary tumor marker. In this context, considering that the majority of patients presented with metastatic or advanced breast cancer, we have opted to monitor all three markers (CEA, CA15-3, and CA-125) to ensure a comprehensive approach and followed the markers in serum as an indicator for response to therapy.

### 3.2. Effects of PECT in cohort of liver involved metastatic cancer patients

As the next step, we were authorized to carry out PECT on the liver, as the most common site of distant metastasis in solid tumors (33), which also plays the leading role in body metabolism and vital functions. Also, the liver is an appropriate targeted organ for PECT due to its proximity to the superficial region of the body (suitable for patch connection and electrical field distribution area).

**Figure 4:**
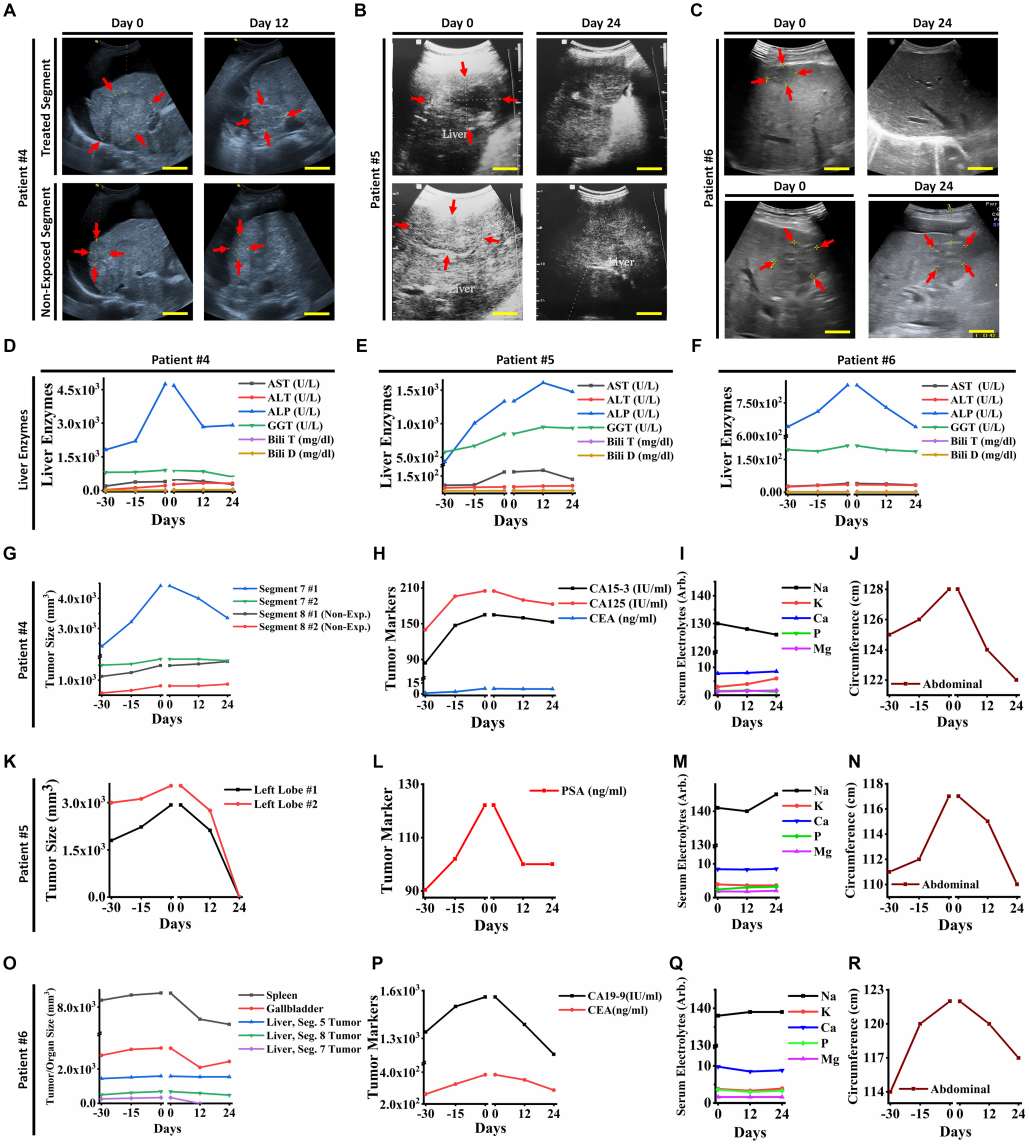
**(A)** Ultrasonography (US) of the liver tumors of patient ID#4. The US showed a reduction in exposed tumor sizes after PECT, while the non-exposed tumors increased in size. **(B)** Ultrasonography images of liver tumors in patient ID#5. The tumors vanished after PECT. **(C)** Ultrasonography images of liver tumor in patient ID#6. It showed complete degradation of the tumor and a decrease in the size of the other one after PECT. **(D,E,F)** The changes in liver enzymes of patient ID#4, #5, and #6 from 30 days before PECT and during PECT. They improved with PECT. **(G)** Liver tumor size graph of patient ID#4. It showed a significant reduction in the size of the tumors in the exposed site, while the tumors in the non-exposed site were stable or increasing in size. **(H)** Cancer-associated antigens of patient ID#4. Their values decreased during PECT, though it was rising 30 days before treatment. **(I)** Serum electrolytes of patient ID#4. They showed no significant changes during PECT. **(J)** Abdominal circumferences alterations of patient ID#4 from 30 days before PECT and during PECT, as an indicator for ascites (34). **(K)** Liver tumor sizes of the patient ID#5. They have been completely degraded after PECT. **(L)** The cancer-associated antigen of patient ID#5. It decreased during PECT, while there was an increasing trend before the PECT. **(M)** Serum electrolytes of patient ID#5 were stable during PECT. **(N)** Abdominal circumference in patient ID#5. It decreased after PECT, while it showed an upward trend before PECT. **(O)** Liver tumor sizes in patient ID#6 were demonstrated 30 days before and during PECT. Tumor in segment 7 vanished completely, and the other ones showed size reduction. **(P)** Cancer-associated antigens in patient ID#6. They were increasing 30 days before PECT, while after the PECT began, they started reduction. **(Q)** Serum electrolytes of patient ID#6 during PECT showed no significant changes. **(R)** Abdominal circumference of patient ID#6. It improved significantly during PECT. The reference intervals of serum biomarkers and blood biochemistry factors are presented in Supplementary Table 3.

Three patients with advanced stages of epithelial carcinoma, including breast cancer (female; ID#4), prostatic adenocarcinoma (male; ID#5), and cholangiocarcinoma (female; ID#6) who had liver metastasis and life-threatening complications with chemo/radio resistances and no indication of surgery, were candidates for receiving PECT on their livers (Figure 4; Supplementary Table 1; Table 1). Each patient had their individual therapeutic protocols, but all had received chemo/radiotherapies for at least a year, with no clinical response. Patients had documented imaging data and liver enzyme tests, suggesting liver metastasis.

They received PECT (482 nC, 30 kV, continuously for 12 days) while hospitalized for better monitoring. As the patients had distinct tumors in various lobes of their livers, the PECT was targeted on individual liver tumors in each patient, and the others were investigated as control (Figure 4; Table 1). After PECT, patients ID#4 and #6 showed clinical improvements in favor of response to this complementary therapy, such as a significant reduction in abdominal circumference and liver heterogeneity as well as the size of the targeted tumors, spleen, and gallbladder (Patient ID#6) (Figures 4A–C,G,K,O). Liver enzyme levels showed a drastic decreasing trend to normal range (Figures 4D–F). Their painkiller usage also reduced remarkably. Besides, the CA19-9 and CEA levels were reduced in patient ID#6, which correlates with the observed reduction in the spleen and gallbladder size, as well as ascites severity (abdominal circumferences) (Figures 4O,P,R). This reduction in spleen and gallbladder size was in line with the improvement of the biliary tract and vascular obstruction, which is in favor of response to therapy. The same decline has been seen in tumor markers and abdominal circumferences (ascites severity) of patient ID#4 (Figures 4H,J).

Observable clinical improvements appeared in patient ID#5 (Table 1). The exposed tumor of the liver was degraded entirely (Figures 4B,K). Drastic reduction in his PSA level and abdominal circumference (Figures 4L,N) were also noticeable. Same as the patients in the breast cancer cohort, none of the patients with liver metastasis showed serum electrolyte perturbation (Figures 4I,M,Q). Additionally, nothing was found correlated with PECT effects against neither cardiac, liver, kidney, spleen, and gastric functions, nor neuronal conduction. These bright observations resulted in the performance of a ten patient-liver cohort (with metastatic liver) that were administered PECT, and improvements in their clinical conditions were observed (Table 1).

We observed that PECT showed considerable controlling and suppressing effects on proliferation and invasive functions of metastatic liver tumor cells. It might be initiated from the affected membrane and cytoskeletal compartments of metastatic liver cells from accumulated electrostatic potential while no dysfunctions were recorded from normal cells of the liver. This might be another way to understand the structural and functional differences between normal liver and malignant metastatic cells (35). They showed entirely different reactions to stimulation with PECs in our study.

### 3.3. PECT in eight patients with different types of metastatic cancer with solid tumors (other than breast and liver metastasis)

Furthermore, eight patients with different types of metastatic cancers (Supplementary Table 1) are discussed here.

**Figure 5:**
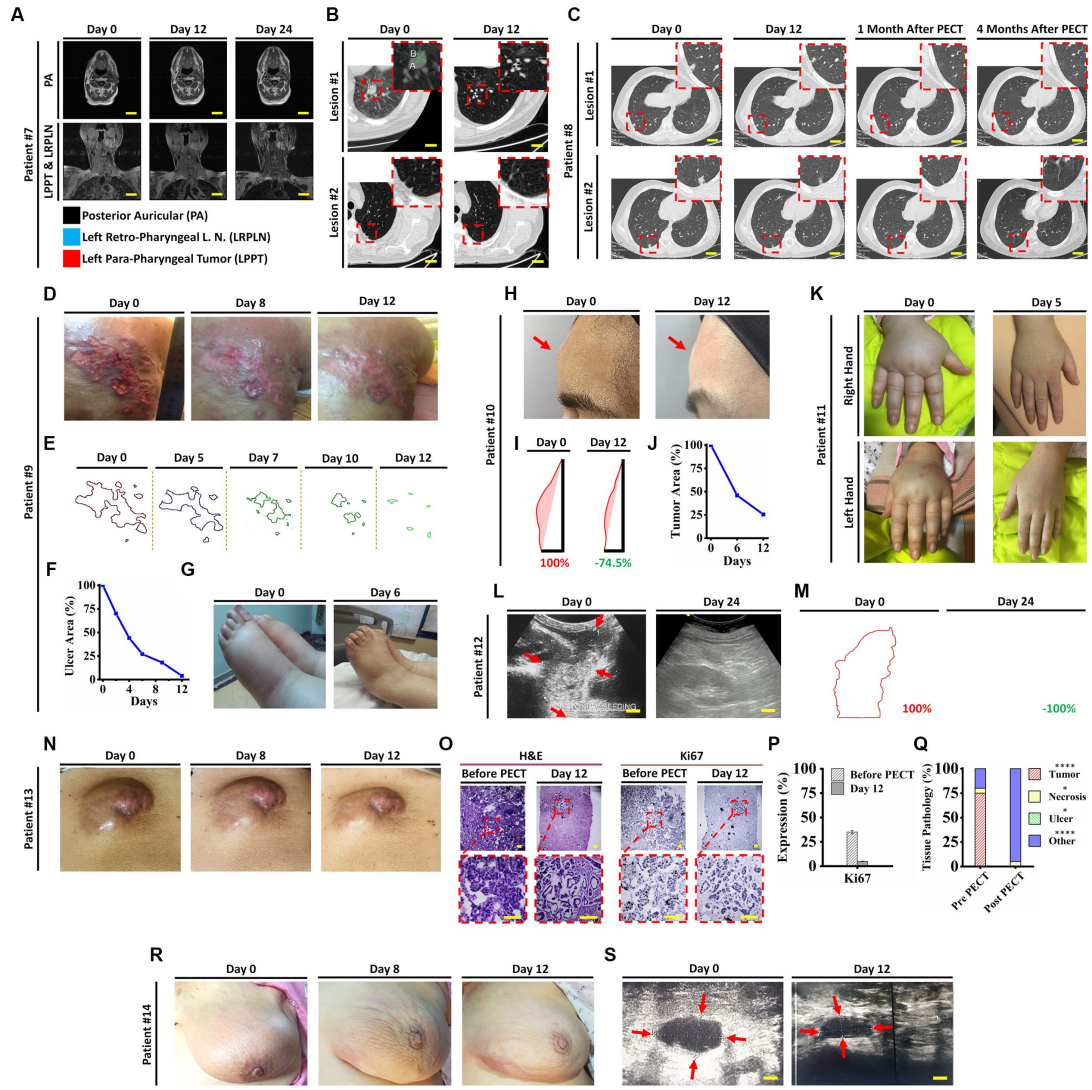
**(A)** Head and neck MRI images of patient ID#7 from beginning to end of the PECT. **(B)** Spiral chest CT scan of patient ID#7 before and after PECT. Inset images demonstrate magnified views of targeted lesions at the left lung lower lobe (Supplementary Movie S1). **(C)** Spiral chest CT scan of patient ID#8 from beginning to end, 1 and 4 months after the PECT. Inset images depict magnified views of targeted lesions at the right lung lower lobe. Obviously, the mentioned lesions were completely degraded, and there was no sign of recurrence after 4 months. Patient ID#9, DCIS patient with 10 years history of unsuccessful therapies with severe symptoms, such as extensive right upper back skin metastasis, left breast triple-negative carcinoma, and metastatic lymph nodes with severe induced lymphedema. **(D)** Optical images of the exposed skin metastasis at the right upper back before, during, and at the end of the PECT. The patient bore severe pain due to an extensive cancer-associated skin ulcer. At the beginning of PECT, the patient’s pain expression drastically decreased, and after 5 days, she stopped using tranquilizers. **(E,F)** Image processing of the cancer-associated skin ulcer from beginning to end of the PECT. Calculations showed a significant reduction of cancerous areas, about~90%. **(G)** The lower limbs’ edema improved significantly, from 4+ pitting edema to 1+, after 6 days of PECT (Supplementary Movie S2). **(H)** Optical images of the forehead tumor of the patient ID#10. **(I,J)** Image processing of the patient’s forehead tumor showed a ~ 75% reduction in size. **(K)** Optical images of the patient ID#11 edematous upper limbs. A significant decrease of mentioned edema occurred after 5 days of PECT. **(L)** Ultrasonography (US) of the epigastric tumor of the patient ID#12. The US showed complete destruction of epigastric tumor after PECT. Scale bars, 1 cm. **(M)** Image processing analysis showed the margins of the epigastric tumor, which completely vanished by PECT. **(N)** optical images of abdominal wall tumor size reduction in patient ID#13. **(O)** H&E and Ki67 immunostaining of the tumor section before and after PECT. Pyknotic nuclei and low density of cancerous cells showed the effect of PECT in both H&E and Ki67 staining. Scale bars, 20 μm. **(P,Q)** Comparative graphs depicted Ki67 expression and pathological tissue components of the tumor section before and after the PECT. The mean of 10 different slides determined the percentage of each component from one biopsied tumor (****p < 0.0001, *p > 0.05, Paired *T*-test). **(R)** Images of patient ID#14 with a significant reduction of tumor size located in the left breast. **(S)** Left breast ultrasonography images showed a remarkable reduction of left inner quadrant edema, upper outer quadrant edema, and 7 O’clock tumor.

**Figure 6:**
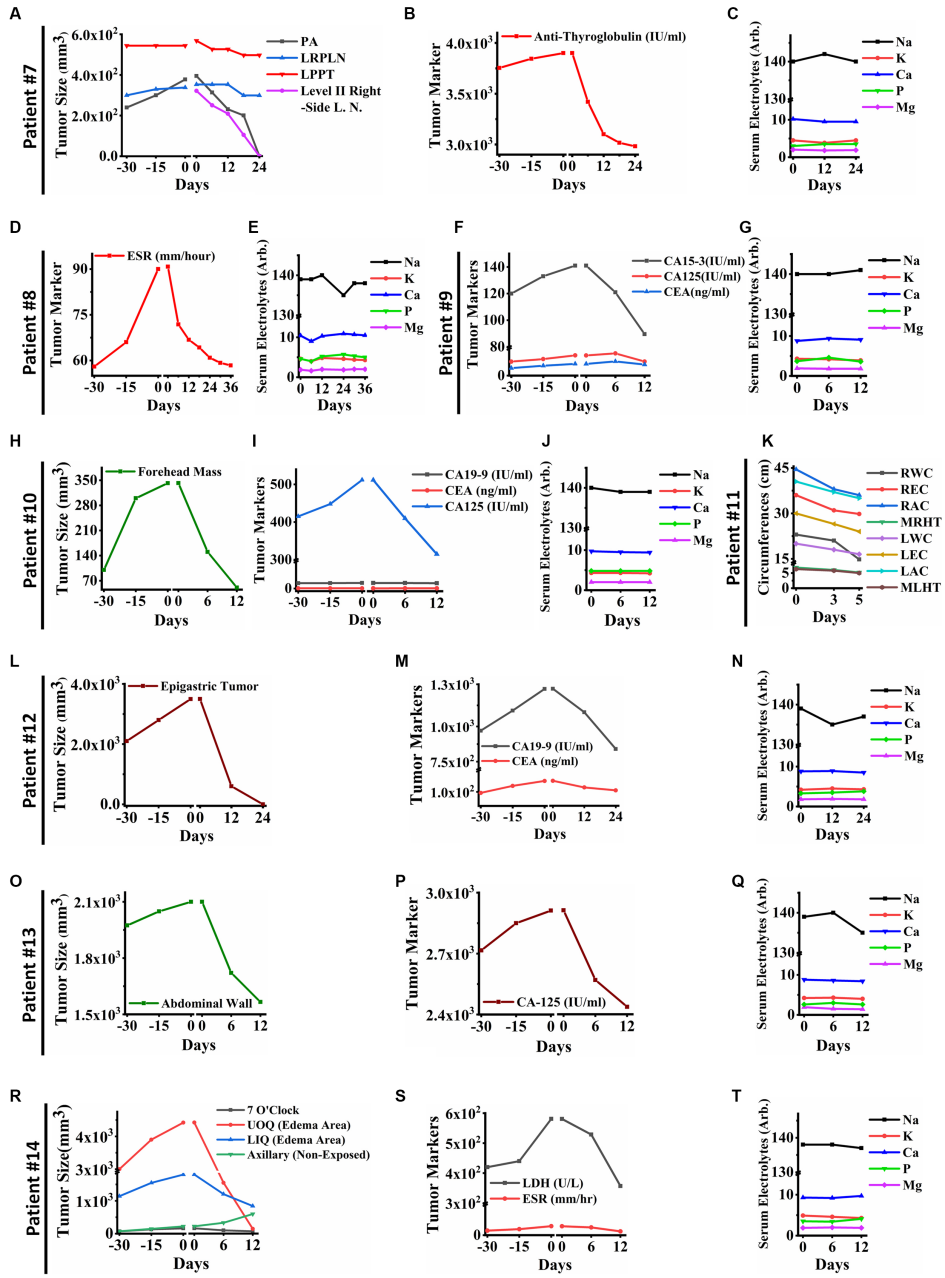
**(A)** Tumor sizes of patient ID#7 from 30 days before PECT and during PECT. Posterior auricular tumor (PA) and lymph node in level II have vanished completely in the right side of the neck. Moreover, Left Retropharyngeal Lymph Node (LRPLN) and Left Parapharyngeal Tumor (LPPT) decreased in size. **(B)** Anti-Thyroglobulin of patient ID#7, as a cancer-associated antigen, decreased significantly while it was increasing before PECT. **(C)** Serum electrolytes of patient ID#7. They did not show significant changes during PECT. **(D)** The cancer-associated antigen of patient ID#8. The Erythrocyte Sedimentation Rate (ESR) of patient ID#8 decreased significantly and reached the normal range. **(E)** Serum electrolytes of the patient ID#8. They were within the normal range during PECT. **(F)** Cancer-associated antigens of patient ID#9. They showed an upward trend before PECT, while they started to decline after electrostatic charge therapy began. **(G)** Serum electrolytes of the patient ID#9. **(H)** Tumor size of the patient ID#10. Forehead mass, which was measured with a caliper, was increasing before the start of the PECT. Although, it showed a significant reduction in size after PECT. **(I)** Cancer-associated antigens of patient ID#10 were demonstrated 30 days before and during PECT. **(J)** Serum electrolytes of the patient ID#10. They did not change significantly during PECT. **(K)** The clinical parameters of the patient ID#11 were measured as follows: Right Wrist Circumference (RWC), Right Elbow Circumference (REC), Right Arm Circumference (RAM), Maximum Right-Hand Thickness (MRHT), Left Wrist Circumference (RWC), Left Elbow Circumference (REC), Left Arm Circumference (RAM), and Maximum Left-Hand Thickness (MRHT). They decreased in size during PECT. **(L)** The size of the epigastric tumor of the patient ID#12. It has entirely vanished after PECT. **(M)** Cancer-associated antigens of the patient ID#12. There was an increasing trend before the PECT, while they started to decrease after the beginning of the PECT. **(N)** Serum electrolytes of the patient ID#12 during PECT. **(O)** The size of the tumor in the abdominal wall of patient #13. It showed a significant size reduction. **(P)** Cancer-associated antigen in patient ID#13. It decreased drastically, while it was increasing before PECT. **(Q)** Serum electrolytes of the patient ID#13. They were stable during PECT. **(R)** Tumor sizes of the patient ID#14. The axillary tumor, which was not exposed to PECT, increased in size. The other tumor sizes decreased. **(S)** Cancer-associated antigens of patient ID#14. They have been significantly reduced during PECT. **(T)** Serum electrolytes of the patient ID#14. They did not change significantly during PECT. The reference intervals of serum biomarkers and blood biochemistry factors are presented in Supplementary Table 3.

Remarkable changes were observed in post PECT state of the patients, such as: reduced size of the posterior auricular (PA), left para-pharyngeal tumor (LPPT), and left retro-pharyngeal lymph node (LRPLN) tumors (Figures 5A, 6A), with degradation of lung metastatic nodules (Figure 5B), and reduced level of antithyroglobulin markers in patient ID#7 (Figure 6B); degradation of lung metastaticvnodules without recurrence in 6-month follow up (Figure 5C) as well as drastic reduction of erythrocyte sedimentation rate (ESR) factor (Figure 6D) in patient ID#8; extensive healing of cancer induced ulcer (Figures 5D,E), drastic reduction in tumor induced oedema (Figures 5F,G), and reduced levels of CA15-3 markers in patient ID#9 (Figure 6F); up to 75% reduction in size of forehead tumor and the tumor area (Figures 5H–J, 6H) with significant reduction in CA-125 marker (an important pulmonary associated neuroendocrine tumor markers [(36, 37); Figure 6I] in patient ID#10; observable reduction of both hands’ oedema in patient ID#11 (Figures 5K, 6K); complete degradation of epigastric tumor in cardiac region (Figures 5L,M, 6L) with drastic reduction in CA19-9 antigen level [main metastasis associated serum marker in gastric cancers (38)] in patient ID #12 (Figure 6M); reduced size of superficial epigastric tumor (Figures 5N, 6O) with almost degraded expression of ki67 in the remaining tumoral lesions (assayed by biopsy before and after PECT, Figures 5O–Q) with sharp reduction in levels of CA-125 enzyme (Figure 6P) in patient ID#13; and finally more than 3 times reduction in breast mass size with diagnosis of lymphoma (Figures 5R,S), severe reduction in breast oedema, and reduced levels of lactate dehydrogenase serum marker [a marker associated with lymphoma progression (39, 40)] (Figures 6R,S) in patient ID#14. The post-treatment expression of caspase-3 in patient ID#13 has been presented in Supplementary Data Sheet.

As with the other cohorts of the current study, neither alterations of serum electrolyte levels (Figures 6C,E,G,J,N,Q,T) nor cardiac dysfunctions were recorded in these patients after PECT. In addition, none of the 41 patients included in the study presented any signs and symptoms in favor of any neuronal disturbance. The patients’ serum electrolytes levels before and after PECT did not change significantly (*p* > 0.05, Supplementary Figure S5). Consequently, the PECT was administered to more than 40 patients with different progressive metastatic cancers in various organs discussed in Supplementary Table 1 and Table 1.

This section highlights the drastic effect of PECT on cancer-associated secreted enzymes and suggests that PECT might reduce metastatic activities of a variety of malignant tumors. This may be related to similar bioelectrical properties of these cells, which show identical dysfunctional responses to positive electrostatic stimulation. In our opinion the role of the membrane, microtubules, and actins as the most electrically active components of the cells, (41–43) could be considered for further investigations.

The current study is the clinical pilot study exploring the effects of positive electrostatic charge on patients with advanced metastatic tumors. We divided the patients into three groups: those with breast tumors (12 patients), liver or metastatic liver tumors (10 patients), and other types of solid tumors (19 patients), received PECT at distinct anatomical sites, such as brain, periumbilical region, epigastric region, neck on the thyroid, flank, groin, eye, and lung. Several considerations warrant attention in future studies. Foremost, future clinical trials should adopt a dual-arm design, comprising a control group (involving placebo or conventional treatment) alongside the PECT group. This arrangement ensures that each patient within the PECT group has an analogous participant within the control group for direct comparison. It is worth emphasizing that patients within each trial should be more consistent in terms of histopathological and tumoral characteristics aspect. The current study includes patients with various tumor types, in which establishing a control group with identical clinical conditions posed considerable challenges. Furthermore, once the efficacy of PECT has been firmly established through clinical trials involving a substantial participant pool, its application can be extended to patients with earlier stages of solid tumors. Of note, PECT may emerge as an attractive alternative for patients who are averse to conventional therapies like chemotherapy due to their associated side effects.

## 4. Conclusion

In summary, we demonstrated the intrinsic ability of pure PECs in destroying malignant tumor with negligible side effects and clinically validated results. PECs was applied to human breast and metastatic liver cancer tumors, and other different types of progressive metastatic solid tumors using patches from the top of the skin. The selective destructive effect was evaluated by radiological imaging, histopathological examinations, and cellular/molecular analyses; As the source of PECs was the Van de Graaff generator, it was completely safe even in voltages higher than 30kv (due to generated currents less than 10 microampere). At least 6 months of follow-up were carried out for the patients, and PECs showed therapeutic outcomes without any side effects. PECT induced no skin burning or neural perturbation and did not change the body’s electrolytic balance (Supplementary Figure S5). Twelve days of continuous exposure to PECT made no uncomfortable feeling in the patients. Palliative therapeutic effects of PECT were started at the first days of therapy by inducing a drastic reduction in the patients’ pain and was concluded by observing reduced tumor size and functions (patient serum and immunohistochemical parameter of their tumor). In the final step, patients with various types of progressive metastatic solid tumors were exposed to optimized suggested protocols of PECT and followed up for at least 6 months. Overall, 41 patients were investigated under PECT, and different quantitative and qualitative analyses indicated remission results.

Impressive therapeutic results observed in this study demonstrate that PECT could be a new complementary therapeutic approach in patients with metastatic malignant tumors combined with other treatments. It may shed new light on bio-electrical hidden factors behind cancer metabolism to achieve safe protocols for cancer treatment.

## Data availability statement

The raw data supporting the conclusions of this article will be made available by the authors, without undue reservation.

## Ethics statement

The studies involving humans were approved by Tehran University of Medical Science Ethical Committee (under Ethical code of IR.TUMS.VCR.REC.1397.354 and clinical study registration No. IRCT20190904044697N2). The studies were conducted in accordance with the local legislation and institutional requirements. Written informed consent for participation in this study was provided by the participants or their legal guardians/next of kin.

## Author contributions

AsZ designed the concept and research, performed most human clinical investigations and statistical analysis, analyzed the data, and wrote and revised the manuscript. FS performed human clinical investigations and statistical analysis, analyzed the data, and wrote and revised the manuscript. FA performed and advised most human clinical investigations, analyzed the data, and revised the manuscript. MF and RA performed and advised the human clinical investigations and revised the manuscript. PH performed histopathological evaluations. AG, MS, ZD, SM, YK, SM-k, and AM performed human clinical investigations. AfZ and SA visited the patients and advised the human clinical investigations. MH reviewed all radiological data. MT performed ultrasonography images of the patients. MST performed ultrasonography images and advised in radiological evaluations. ZSM, MN, and MEA advised the human clinical investigations. ME performed electrostatic charge generators’ maintenance and human clinical investigations. MY revised the manuscript. MA designed the concept and research, analyzed the data, and wrote and revised the manuscript. All authors contributed to the article and approved the submitted version.
