# Supplementary material for: A human pilot study on positive electrostatic charge effects in solid tumors of the late-stage metastatic patients
Source: Front Med (Lausanne). 2023 Oct 17;10:1195026. doi: 10.3389/fmed.2023.1195026 (PMC10616960; doi:10.3389/fmed.2023.1195026)
Supplement: Supplementary file 1 [file Table_1.docx]

***Supplementary Table 1,*** *Principal demographic characteristics of the patients, the origin of disease, metastatic sites, PECT treated area, and the clinical manifestations.*

| Patient ID | Sex | Age (Years) | Ethnicity | Origin of disease | Metastatic Sites | Previous Treatment | PECT Site | Clinical Manifestations |
| --- | --- | --- | --- | --- | --- | --- | --- | --- |
| 1 | F | 83 | Persian | Metastatic Invasive Ductal Carcinoma of the Breast | Bone lesions Axillary nodules | Low-dose Chemotherapy  (No surgery due to medical condition and advanced age) | Left Breast and left axillary | -Anorexia -Sleeping Difficulty -Severe left upper limb pain -Severe edema in left Breast and left upper limb |
| 2 | F | 42 | Kurd | Metastatic Invasive Ductal Carcinoma of the Breast | Breast Axillary nodules | Breast-conserving surgery  Standard Chemotherapy  Standard Radiotherapy | Left Breast | -Left breast mass -Edema, pain, and discomfort in the left Breast |
| 3 | F | 58 | Persian | Metastatic Invasive Ductal Carcinoma of the Breast | Lung Axillary nodules | No surgery (Not consented)  Chemotherapy (2 Course, decline to continue)  Different Herbal treatment | Right Breast | -Cough -Anorexia -Sleeping Difficulty -Pain in right Breast -Dyspnea NYHA IV -Stiffness and edema of right Breast -Nodules and masses in right Breast |
| 4 | F | 50 | Persian | Metastatic Invasive Ductal Carcinoma of the Breast | Liver Bone lesions | Modified Radical Mastectomy  Chemotherapy | Liver | -Ascites -Anorexia -Sleeping Difficulty -General weakness -Chronic Abdominal Pain |
| 5 | M | 60 | Turk | Metastatic Prostate Cancer | Liver Bone lesions | Radical Prostatectomy  Orchidectomy  Radiotherapy | Liver | -Anorexia -General Weakness -Chronic Abdominal Pain -Sleeping and defecation difficulty |
| 6 | M | 67 | Kurd | Metastatic cholangiocarcinoma of Liver | Liver | Chemotherapy (discontinued due to side effects) | Liver | -Ascites -Anorexia -Epigastric pain -General weakness -Sleeping difficulty -Obstruction of bile ducts |
| 7 | F | 64 | Persian | Metastatic Papillary Thyroid Carcinoma | Lung Neck Lymphadenopathies | Radical Thyroidectomy  Chemotherapy  Radioactive Iodine Therapy | Neck and lung | -Dysphagia -Neck masses  -Hoarseness |
| 8 | M | 14 | Turk | Metastatic Small cell Carcinoma of the kidney | Lung | Nephrectomy  Standard Chemotherapy | Both Lung Lobes | - |
| 9 | F | 54 | Persian | Metastatic Invasive Ductal Carcinoma of the Breast | The skin of the right back, right hand, and site of mastectomy | Modified radical Mastectomy  Multiple Chemotherapy Regimens  Radiotherapy | The wound on the right Breast The wound on the right upper back | -Anorexia -Sleeping Difficulty -Discharge of wounds -Severe pain in the location of ulcers (Breast and back) -Open non-healing wound at the site of mastectomy and upper back on the skin |
| 10 | F | 31 | Persian | Small cell neuroendocrine carcinoma | Skin Lung Liver | Extensive Chemotherapy | Frontal mass | -Cough -Anorexia -General weakness -Diffuse skin masses, including frontal mass |
| 11 | F | 50 | Kurd | Metastatic Medullary Thyroid Carcinoma | Liver Brain Bone lesions Multiple axillary lymphadenopathies | Subtotal Thyroidectomy  Radiation therapy | Left and Right axilla | Anorexia General weakness Upper limbs edema Chronic pain in upper limbs |
| 12 | F | 64 | Arab | Metastatic Gastric Adenocarcinoma | Epigastric Mass Peritoneal seeding Anastomotic recurrence | Total gastrectomy and esophagojejunostomy  Chemotherapy | Epigastric region | -Anorexia -Dyspepsia -Epigastric pain -General Weakness -Sleeping difficulty |
| 13 | F | 66 | Persian | Metastatic Serous Carcinoma of Ovary | Peritoneal Carcinomatosis Metastatic Epigastric Mass | Salpingo-oophorectomy  Chemotherapy | Epigastric Wall | -Anorexia -General weakness -Sleeping difficulty -Pain of epigastric mass -Open non-healing wound at the site of hysterectomy on the skin |
| 14 | F | 84 | Lur | Breast Diffuse Large B-Cell Lymphoma | Breast Axillary nodules Diffuse Lymphadenopathy | Low-dose chemotherapy (advanced age and medical condition) | Left Breast | -Anorexia -General weakness -Edema of left Breast -Pain and discomfort of left Breast |
| 15 | M | 10 | Persian | Metastatic Papillary Thyroid Carcinoma | Lung Neck lymphadenopathies | Subtotal Thyroidectomy  Radioactive Iodine Therapy  Chemotherapy | Lungs | -Anorexia -General weakness -Sleeping difficulties -Dyspnea NYHA IV |
| 16 | M | 20 | Kurd | Metastatic Liposarcoma | Lung | Surgery  Chemotherapy  Lung Metastasectomy | Lungs | -Cough -Anorexia -General weakness -Sleeping difficulty |
| 17 | F | 28 | Persian | Metastatic Wilms' tumor of the kidney | Lungs | Nephrectomy  Chemotherapy | Lungs | - |
| 18 | F | 29 | Tajik | Invasive Ductal Carcinoma of the Breast | No Metastasis | Breast-conserving Surgery  Chemotherapy | Left breast mass | -Anorexia -Left breast edema -General weakness -Huge left breast mass -Left breast and left breast mass pain |
| 19 | M | 33 | Persian | Metastatic myoepithelial carcinoma | Lung  Soft tissue mass in the upper left back | Surgery  Chemotherapy | Left upper back | -Cough -Anorexia -Inability to walk -Sleeping difficulty -Dyspnea NYHA IV -Left upper back mass -Inability to sleep in the supine position -Severe pain in left upper back mass and inability to lie down on it |
| 20 | F | 34 | Persian | Metastatic Papillary Thyroid Carcinoma | Lung Neck lymphadenopathies | Total Thyroidectomy  Radioactive iodine therapy  Reactive Iodine Therapy | Neck | -Dysphagia -Hoarseness |
| 21 | F | 35 | Persian | Metastatic Invasive Ductal Carcinoma of the Breast | Skin | Modified Radical Mastectomy  Chemotherapy | Right Breast | -Anorexia -General weakness -Sleeping difficulties -Inability to walk alone -Expanding wound of right Breast with bleeding and discharge |
| 22 | F | 39 | Persian | Metastatic Invasive Ductal Carcinoma of the Breast | Skin | Modified Radical Mastectomy  Chemotherapy | Right Breast and skin of the Breast | -Erythema of the skin and site of mastectomy |
| 23 | F | 42 | Persian | Metastatic Ovarian Cancer | Cerebellum Peritoneal Carcinomatosis | Salpingo-oophorectomy  Chemotherapy | Periumbilical region | -Anorexia -General weakness -Sleeping difficulties -Nausea and vomiting -Inability to walk alone |
| 24 | F | 47 | Lur | Metastatic Colon Adenocarcinoma | Liver Spleen Pelvic cavity Periumbilical mass Peritoneal carcinomatosis | Multiple surgeries  Multiple Chemotherapy Courses | Liver Periumbilical region | -Ascites -Anorexia -General weakness -Sleeping difficulty -Severe Abdominal Pain |
| 25 | F | 49 | Persian | Metastatic Invasive Ductal Carcinoma of the Breast | Lung Skin | Modified Radical Mastectomy  Chemotherapy | Left Breast and left upper back | -Cough -Anorexia -Inability to walk -Sleeping Difficulty -Dyspnea NYHA IV -Discharge of wounds -Severe pain in the location of ulcers (Breast and back) -Open non-healing wound at the site of mastectomy and upper back on the skin |
| 26 | F | 50 | Persian | Metastatic Invasive Ductal Carcinoma of the Breast | Brain Liver Bone Lesions | Breast-Conserving surgery  Radiotherapy  Multiple Chemotherapy Regimens | Liver | -Anorexia -Sleeping Difficulty -General weakness -Lower back and abdominal Pain |
| 27 | F | 50 | Persian | Metastatic Invasive Ductal Carcinoma of the Breast | Liver Bone lesions Supraclavicular Lymphadenopathy | Modified Radical Mastectomy  Chemotherapy (discontinued due to side effects) | Liver | -Anorexia -Sleeping Difficulty -General weakness -Pain in right scapula and abdomen |
| 28 | F | 50 | Persian | Invasive Ductal Carcinoma of the Breast | - | Bilateral Modified Radical Mastectomy (declined any adjuvant therapy) | The wound of the Right Breast (site of mastectomy) | -Right Breast -Open non-healing wound at the site of mastectomy on the skin |
| 29 | F | 50 | Persian | Metastatic ovarian cancer | Peritoneal cavity Peritoneal carcinomatosis | Multiple courses of chemotherapy | Periumbilical | -Anorexia -General weakness -Sleeping difficulty -Chronic abdominal pain |
| 30 | M | 55 | Kurd | Squamous cell carcinoma of the cornea | - | Surgical Excision  Topical Chemotherapy | Right eye (cornea) | -General weakness -Visual impairment |
| 31 | M | 60 | Persian | Metastatic Hemangiopericytoma | Soft tissue masses in the groin, flank, legs, head, pelvic cavity, etc. | Surgery  Chemotherapy | Left groin | -Anorexia -General weakness -Sleeping difficulty -Right groin pain -Right lower limb paresthesia -pelvic cavity pain -Severe pain in lower back, groin, and legs |
| 32 | M | 60 | Persian | Glioblastoma | - | Surgery  Radiotherapy  Chemotherapy | Brain | -Anorexia -General weakness -Sleeping difficulties -Loss of consciousness |
| 33 | M | 66 | Persian | Metastatic Liposarcoma | Soft tissue masses in the left flank and legs | Surgery on left knee  Standard Chemotherapy  Standard Radiotherapy | Left Flank | -Anorexia -Left leg mass -Sleeping difficulty -General weakness -Pain in the left flank and left leg |
| 34 | F | 68 | Turk | Invasive Ductal Carcinoma of the Breast | Skin | Modified Radical Mastectomy  Chemotherapy | The wound on the Left Breast | -Anorexia -General weakness -Sleeping difficulties -Inability to walk alone -Expanding wound of left Breast with bleeding and discharge |
| 35 | M | 70 | Persian | Metastatic colon adenocarcinoma | Lung Liver | Surgery  Multiples Chemotherapy Courses | Liver | -General weakness |
| 36 | M | 70 | Persian | Metastatic Colon adenocarcinoma | Liver | Surgery  Multiple Chemotherapy Courses | Liver | -Anorexia -General weakness -Sleeping difficulties -Inability to walk alone -Tenderness and pain in the right upper quadrant |
| 37 | M | 72 | Turk | Metastatic Gastric B-cell lymphoma | Liver Gastric lymphadenopathies | Chemotherapy (progressed to liver metastasis under chemotherapy) | Liver | -Anorexia -Inability to walk -General weakness -Sleeping difficulty |
| 38 | M | 75 | Persian | Nasopharyngeal carcinoma | - | Radiation Therapy  Chemotherapy | Neck | -Anorexia -Dysphagia -inability to talk -Hoarseness of voice -General weakness -Sleeping difficulties -Inability to walk alone |
| 39 | F | 76 | Persian | Invasive Ductal Carcinoma of the Breast | Lungs Mastectomy site | Modified Radical Mastectomy  Chemotherapy | Right Breast  mastectomy site | -Anorexia -General weakness -Sleeping difficulties -Inability to walk alone -Tenderness and pain in right Breast |
| 40 | F | 78 | Lur | Metastatic Colon adenocarcinoma | Liver | Surgery  Standard Chemotherapy | Liver | -Anorexia -Abdominal Pain -Sleeping difficulty -General weakness |
| 41 | F | 82 | Turk | Invasive Ductal Carcinoma of the Breast | - | Low-dose chemotherapy (advanced age) | Left Breast | -Breast masses -Pain in Left Breast |
