# Supplementary material for: A human pilot study on positive electrostatic charge effects in solid tumors of the late-stage metastatic patients
Source: Front Med (Lausanne). 2023 Oct 17;10:1195026. doi: 10.3389/fmed.2023.1195026 (PMC10616960; doi:10.3389/fmed.2023.1195026)
Supplement: Supplementary file 2 [file Table_2.docx]

***Supplementary Table 2,*** *Detailed characteristics of each patient *PECT courses, sites, voltage, and charge*

| Patient ID | PECT  Site | Applied Voltage (kV)  (± 5Kv) | Positive Electrostatic Charge (nC)  (± 80 nC) | *Total Course of PECT | Total Days of PECT | Timeline | Hospitalization |
| --- | --- | --- | --- | --- | --- | --- | --- |
| 1 | Left Breast and left axillary | 30 | 480 | 1 | 12 | Continuously | Hospitalized |
| 2 | Left Breast | 30 | 480 | 1 | 12 | Continuously | Hospitalized |
| 3 | Right Breast | 30 | 480 | 1 | 12 | Continuously | Hospitalized |
| 4 | Liver | 30 | 480 | 2 | 24 | Continuously | Hospitalized |
| 5 | Liver | 30 | 480 | 2 | 24 | Continuously | Hospitalized |
| 6 | Liver | 30 | 480 | 2 | 24 | Continuously | Hospitalized |
| 7 | Neck and lung | 30 | 480 | 3 | 36 | Continuously | Hospitalized |
| 8 | Both Lung Lobes | 30 | 480 | 1 | 12 | Continuously | Hospitalized |
| 9 | Wound of right breast Wound of right upper back | 30 | 480 | 1 | 12 | Continuously | Hospitalized |
| 10 | Frontal mass | 30 | 480 | 1 | 12 | Continuously | Hospitalized |
| 11 | Left and Right axilla | 30 | 480 | 1 | 5 | Continuously | Hospitalized |
| 12 | Epigastric region | 30 | 480 | 2 | 24 | Continuously | Hospitalized |
| 13 | Epigastric Wall | 30 | 480 | 1 | 12 | Continuously | Hospitalized |
| 14 | Left Breast | 30 | 480 | 1 | 12 | Continuously | Hospitalized |
| 15 | Lungs | 0 | 0 | 0 | 0 | - | Hospitalized |
| 16 | Lungs | 30 | 480 | 2 | 24 | Continuously | Hospitalized |
| 17 | Lungs | 30 | 480 | 1 | 12 | Continuously | Hospitalized |
| 18 | Left breast mass | 30 | 480 | 1 | 12 | Continuously | Hospitalized |
| 19 | Left upper back | 30 | 480 | 1 | 12 | Continuously | Hospitalized |
| 20 | Neck | 30 | 480 | 2 | 24 | Continuously | Hospitalized |
| 21 | Right breast | 0 | 0 | 0 | 0 | - | Hospitalized |
| 22 | Right Breast and skin of the breast | 30 | 480 | 1 | 12 | Continuously | Hospitalized |
| 23 | Periumbilical region | 0 | 0 | 0 | 0 | - | Hospitalized |
| 24 | Liver Periumbilical region | 30 | 480 | 3 | 36 | Non- continuously | Hospitalized |
| 25 | Left breast and left upper back | 30 | 480 | 2 | 24 | Continuously | Hospitalized |
| 26 | Liver | 30 | 480 | 2 | 24 | Continuously | Hospitalized |
| 27 | Liver | 30 | 480 | 2 | 24 | Continuously | Hospitalized |
| 28 | Wound of Right Breast (site of mastectomy) | 30 | 480 | 1 | 12 | Continuously | Hospitalized |
| 29 | Periumbilical region | 30 | 480 | 1 | 12 | Continuously | Hospitalized |
| 30 | Right eye (cornea) | 30 | 480 | 1 | 12 | Continuously | Hospitalized |
| 31 | Left groin | 30 | 480 | 1 | 12 | Continuously | Hospitalized |
| 32 | Brain | 0 | 0 | 0 | 0 | - | Hospitalized |
| 33 | Left Flank | 30 | 480 | 1 | 12 | Continuously | Hospitalized |
| 34 | Skin | 30 | 480 | 1 | 12 | Continuously | Hospitalized |
| 35 | Liver | 30 | 480 | 2 | 24 | Continuously | Hospitalized |
| 36 | Liver | 30 | 480 | 2 | 24 | Continuously | Hospitalized |
| 37 | Liver | 30 | 480 | 2 | 24 | Continuously | Hospitalized |
| 38 | Neck | 0 | 0 | 0 | 0 | - | Hospitalized |
| 39 | Right breast  mastectomy site | 30 | 480 | 1 | 12 | Continuously | Hospitalized |
| 40 | Liver | 30 | 480 | 2 | 24 | Continuously | Hospitalized |
| 41 | Left breast | 30 | 480 | 1 | 12 | Continuously | Hospitalized |

*One course of PECT defined as receiving positive electrostatic charge from the metallic patch on the tumor site, continuously for 12 days, except for daily routine
