# Supplementary material for: A human pilot study on positive electrostatic charge effects in solid tumors of the late-stage metastatic patients
Source: Front Med (Lausanne). 2023 Oct 17;10:1195026. doi: 10.3389/fmed.2023.1195026 (PMC10616960; doi:10.3389/fmed.2023.1195026)
Supplement: Supplementary file 3 [file Table_3.docx]

| Test | Sex and Age Adjacent | | Reference Interval | Unit |
| --- | --- | --- | --- | --- |
| Na | Male and Female | | 135-146 | mmol/L |
| K | Male and Female | | 3.5-5.1 | mmol/L |
| Mg | Male and Female | | 1.6-2.3 | mg/dl |
| Ca | Male and Female | | 8.4-10.5 | mg/dl |
| P | Male and Female | | 2.6-4.5 | mg/dl |
| AST(SGOT) | Male | | <40 | U/L |
|  | Female | | <31 |  |
| ALT(SGPT) | Male | | <45 | U/L |
|  | Female | | <34 |  |
| ALP | Male | | <270 | U/L |
|  | Female | | <240 |  |
| y-GT | Male | | 9-52 | U/L |
|  | Female | | 9-35 |  |
| ESR | Male | 17-51 year | <10 | mm/hr |
|  |  | 51-61 year | <12 |  |
|  |  | 61-70 year | <14 |  |
|  |  | 70-150 year | <30 |  |
|  | Female | 17-51 year | <12 |  |
|  |  | 51-55 year | <19 |  |
|  |  | 55-70 year | <20 |  |
|  |  | 70-150 year | <35 |  |
|  | Male and Female | 0-2 day | 0-4 |  |
|  |  | 2day-17 year | <10 |  |
| PSA | Male | | <4 | ng/ml |
| LDH | Male and Female | | 200-450 | U/L |
| CRP | Male and Female | | <10 | mg/L |
| CEA | Male and Female | | <5 | ng/ml |
| CA19-9 | Male and Female | | <37 | IU/ml |
| CA15-3 | Male and Female | | <30 | IU/ml |
| CA125 | Male and Female | | <35 | IU/ml |
| αFP | Male and Female | | <7.25 | IU/ml |
| hCG | Male and Female | | <5 | IU/ml |

***Supplementary Table 3****,* *the reference intervals of serum biomarkers and blood biochemistry factors (enzymes and electrolytes).*
