# Supplementary material for: A human pilot study on positive electrostatic charge effects in solid tumors of the late-stage metastatic patients
Source: Front Med (Lausanne). 2023 Oct 17;10:1195026. doi: 10.3389/fmed.2023.1195026 (PMC10616960; doi:10.3389/fmed.2023.1195026)
Supplement: Supplementary file 6 [file Data_Sheet_1.docx]

Supplementary Information

**A Human Pilot Study on Positive Electrostatic Charge Effects in Solid Tumors of The Late-Stage Metastatic Patients**

Ashkan Zandi^a,b^, Fatemeh Shojaeian^a,c^, Fereshteh Abbasvandi^d^, Mohammad Faranoush^e,f^, Robab Anbiaee^g^, Parisa Hoseinpour^a,h^, Ali Gilani^a^, Mohammad Saghafi^a^, Afsoon Zandi^i^, Meisam Hoseinyazdi^j^, Zahra Davari sh.^a^, Seyyed Hossein Miraghaie^a^, Mahtab Tayebi^d^, Morteza Sanei Taheri^k^, S. Mehdi Samimi Ardestani^l^, Zahra Sheikhi-Mobarakeh^m^, Mohammad Reza Nikshoar^n^, Mohammad Hossein Enjavi^a,b^, Yasin Kordehlachin^a^, S. M. Sadegh Mousavi-kiasary^a^, Amir Mamdouh^a^, Mohammad Esmaeil Akbari^o^, Masud Yunesian^p,q^, Mohammad Abdolahad^a,b,r,s*^

^a^ Nano Electronic Center of Excellence, Nanobioelectronic Devices Lab., Cancer Electronics Research Group, School of Electrical and Computer Eng., Faculty of Engineering, University of Tehran, P.O. Box: 14395-515, Tehran, Iran.

^b^ Nano Electronic Center of Excellence, Nanoelectronics and Thin Film Lab., School of Electrical and Computer Eng.,Faculty of Engineering, University of Tehran, P.O. Box: 14395-515, Tehran, Iran.

^c^ School of Medicine, Shahid Beheshti University of Medical Sciences, P.O. Box: 19615-1179, Tehran, Iran.

^d^ ATMP Department, Breast Cancer Research Center, Motamed Cancer Institute, ACECR, P.O. Box: 1517964311, Tehran, Iran.

^e^ Pediatric Growth and Development Research Center, Institute of Endocrinology and Metabolism, Iran University of Medical Sciences, P.O. Box: 1996713883, Tehran, Iran.

^f^ Cardio-Oncology Research Center, Rajaie Cardiovascular Medical & Research Center, Iran University of Medical Sciences, P.O. Box: 1996911151, Tehran, Iran.

^g^ Department of Radiation Oncology, Imam Hossein Hospital, Shahid Beheshti University of Medical Sciences, P.O. Box: 19615-1179, Tehran, Iran.

^h^ SEPAS Pathology Laboratory, P.O.Box: 1991945391, Tehran, Iran.

^i^ Department of Otolaryngology, Head & Neck Surgery, Taleghani Hospital, Shahid Beheshti University of Medical Sciences, P.O. Box: 19615-1179, Tehran, Iran.

^j^ Medical Imaging Research Center, Shiraz University of Medical Sciences, P.O. BOX: 71348-14336, Shiraz, Iran.

^k^ Department of Radiology, Shohada Hospital, Shahid Beheshti University of Medical Sciences, P.O. Box: 1445613131, Tehran, Iran.

^l^ Department of Psychiatry, Behavioral Sciences Research Center, Imam Hossein Hospital, Shahid Beheshti University of Medical Sciences, P.O. Box: 19615-1179, Tehran, Iran.

^m^ Quality of Life Department, Breast Cancer Research Center, Motamed Cancer Institute, ACECR, P.O. Box: 1517964311, Tehran, Iran.

^n^ Department of Gastroenterology Surgery, Taleghani Hospital, Shahid Beheshti University of Medical Sciences, P.O. Box: 19615-1179, Tehran, Iran.

^o^ Cancer Research Center, Shahid Beheshti University of Medical Sciences, P.O. Box: 1989934148, Tehran, Iran.

^p^ Department of Environmental Health Engineering, School of Public Health, Tehran University of Medical Sciences, Tehran, Iran

^q^ Department of Research Methodology and Data Analysis, Institute for Environmental Research, Tehran University of Medical Sciences, Tehran, Iran

^r^ Cancer Institute, Imam-Khomeini Hospital, Tehran University of Medical Sciences, P.O. BOX 13145-158, Tehran, Iran

^s^ UT&TUMS Cancer Electrotechnique Research Center, YAS Hospital, P.O. Box 1598718311, Tehran, Iran

* Corresponding author: [m.abdolahad@ut.ac.ir](mailto:m.abdolahad@ut.ac.ir), [abdolahad@sina.tums.ac.ir](mailto:abdolahad@sina.tums.ac.ir)

To investigate the electrostatic field distribution in the human body, the field distribution has been simulated using a 3D electromagnetic analysis software by finite element method (FEM). The patch is located on the breast, liver, and neck to explore the electrostatic field (EF) in our referred patients' three most studied sites. The studied model is a 40 YO woman with 176 cm height and 79 kg weight which is the most similar model to our female patients.

Field investigation in breast tissue depicts high levels of EF distribution in it, which assured us about the breast tumors' exposure uniformly. Axial sections demonstrate almost the same amounts of EF in the breast tissue as the surface of the patch. Accordingly, the field investigations on the liver (Abdomen and pelvic) and neck illustrated high amounts of EF in depth which covered and exposed all the tumors uniformly in the liver and neck.

The applied electric charge is determined using the following calculation:

The electric potential of a point charge is:

$$V=\frac{\mathrm{kq}}{r}\to q=\frac{\mathrm{rV}}{k},$$

where q is the point charge, r is the distance from the charge, and k is a constant equal to

$$k=8.99\times{10}^{9} \frac{N.m^{2}}{C^{2}}.$$

Therefore, if we have the electric potential (using an electrostatic charge meter, MEECH, 983V2) and the distance from the charge (as we assume the center of the sphere on the top of the Van de Graff generator to be the location of the total charge and the radius of the sphere to be the distance to solve the equation without any complexity), the total charge will be:

$$q= \frac{\mathrm{rV}}{8.99 \times{10}^{9}}.$$

if *V=1 kV*, and *r= 14 cm*, then *q=15.57 nC = ~ 16 nC*

Hence, if ***V= 30 kV***, then ***q= 30×16 nC= 480 nC***.


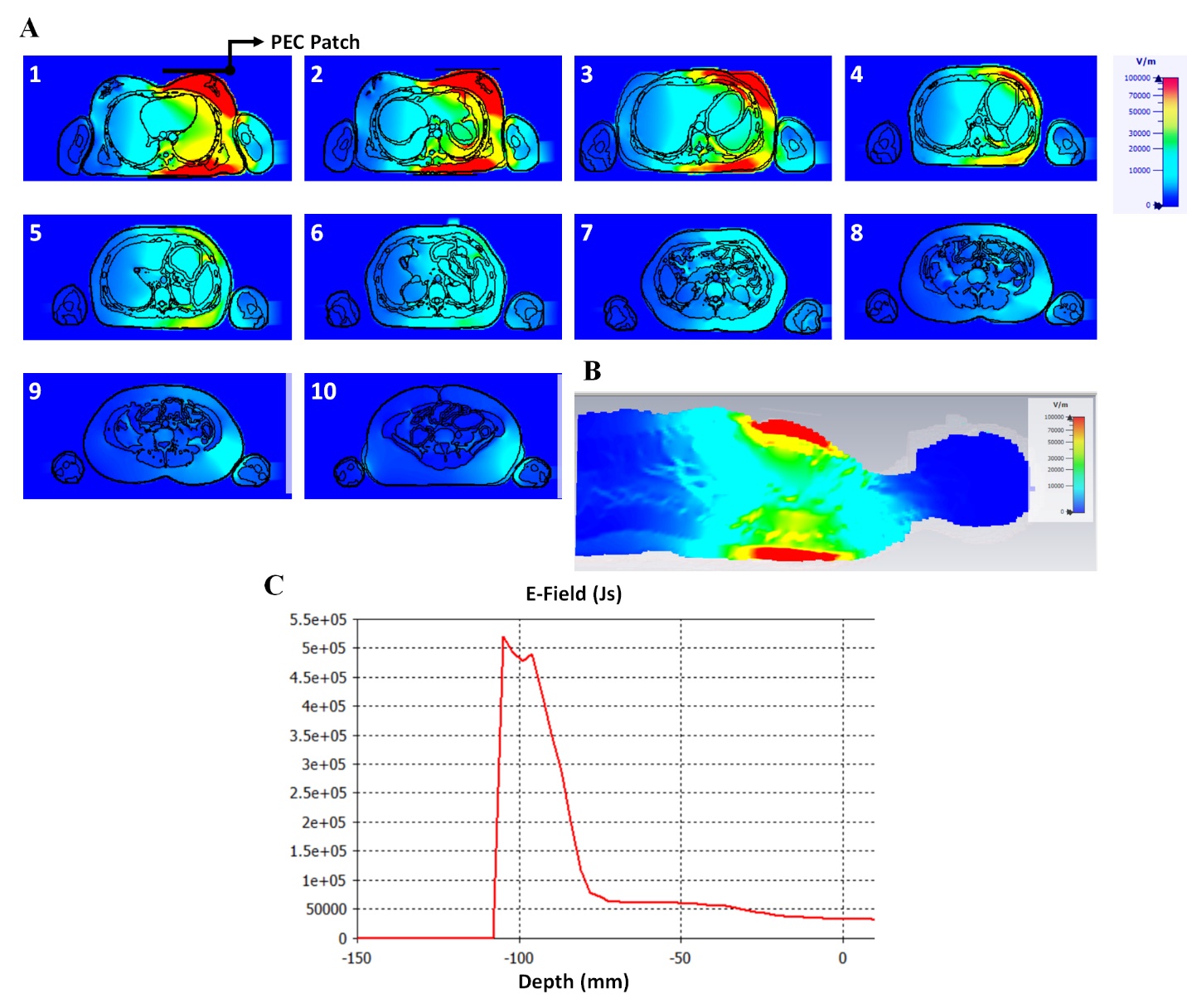


**Figure S1**, (**A**) Axial sections of the simulated induced electrostatic field by locating the electrostatic patch on top of the patient skin. The simulations show the almost uniform distribution of the electrostatic field (EF) in the breast tissue. (**B**) A sagittal view of the field simulation shows the good distribution of the EF in the breast tissue. (**C**) Quantitative graph of EF distribution by depth in the breast and thorax.


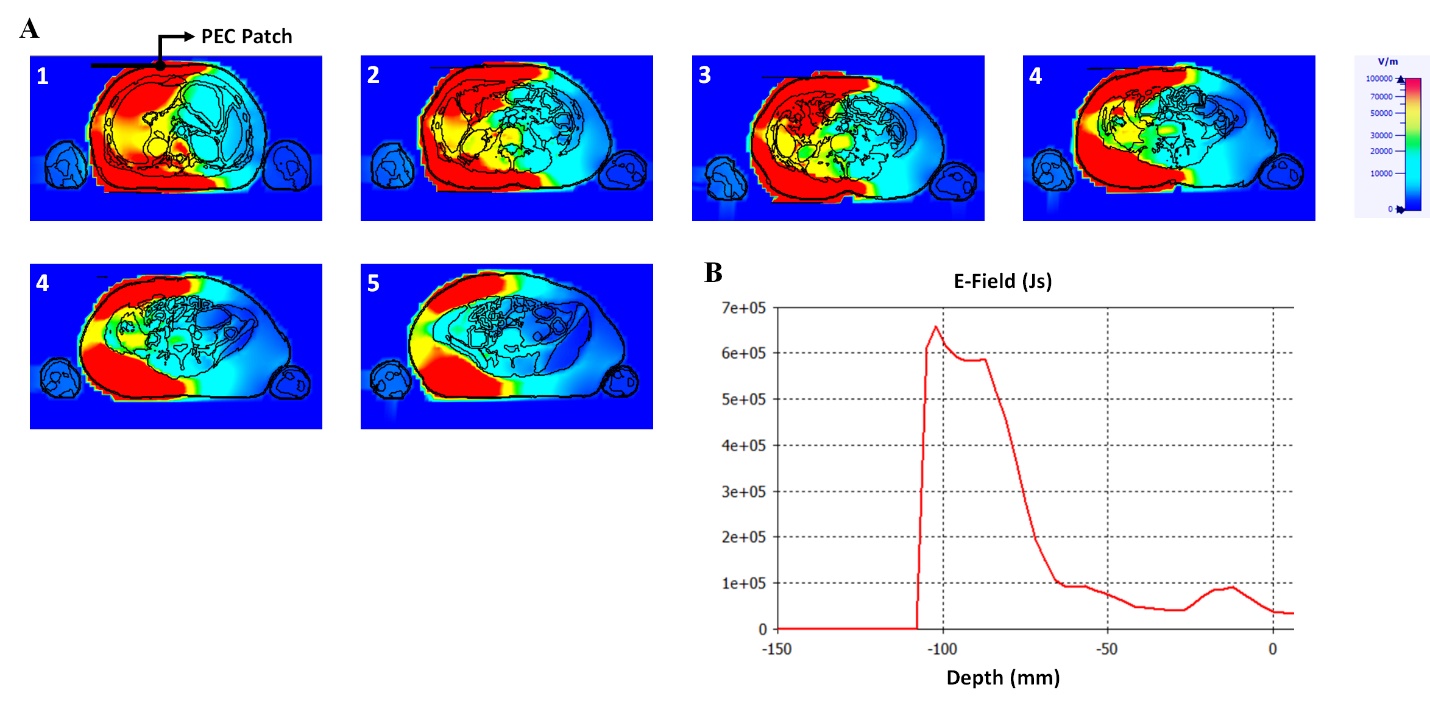


**Figure S2**, (**A**) Axial sections of the simulated induced electrostatic field by locating the electrostatic patch on top of the patient’s liver. Uniform distribution of the electrostatic field (EF) in the liver proves the liver tumors' same exposure. (**B**) Quantitative graph of EF distribution by depth in the liver and abdomen.


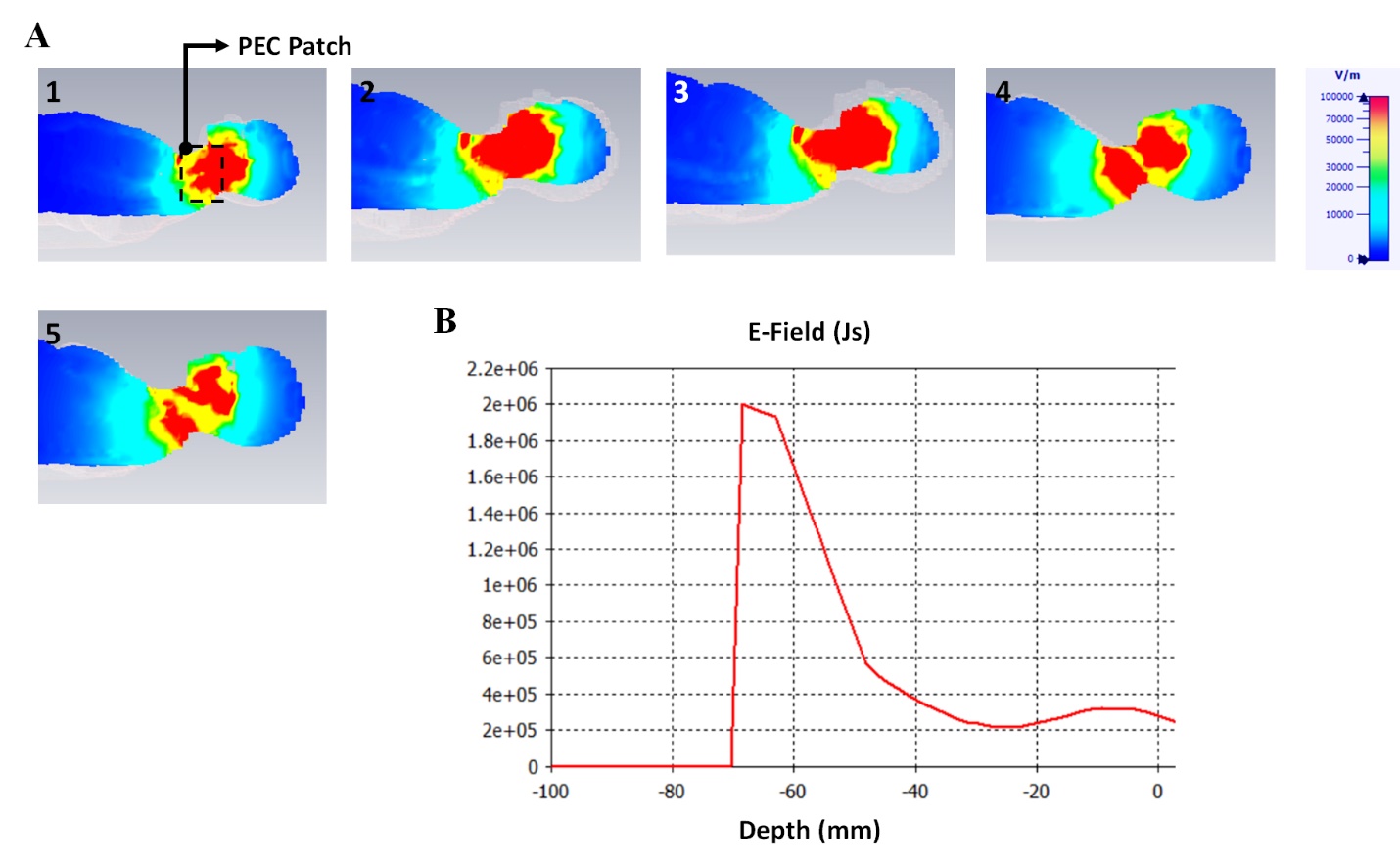


**Figure S3**, (**A**) Axial sections of the simulated induced electrostatic field by locating the electrostatic patch on the right side of the patient’s neck. As it is obvious, the EF covers the neck and will expose all the tumors in the area. (**B**) Quantitative graph of EF distribution by depth in the neck.


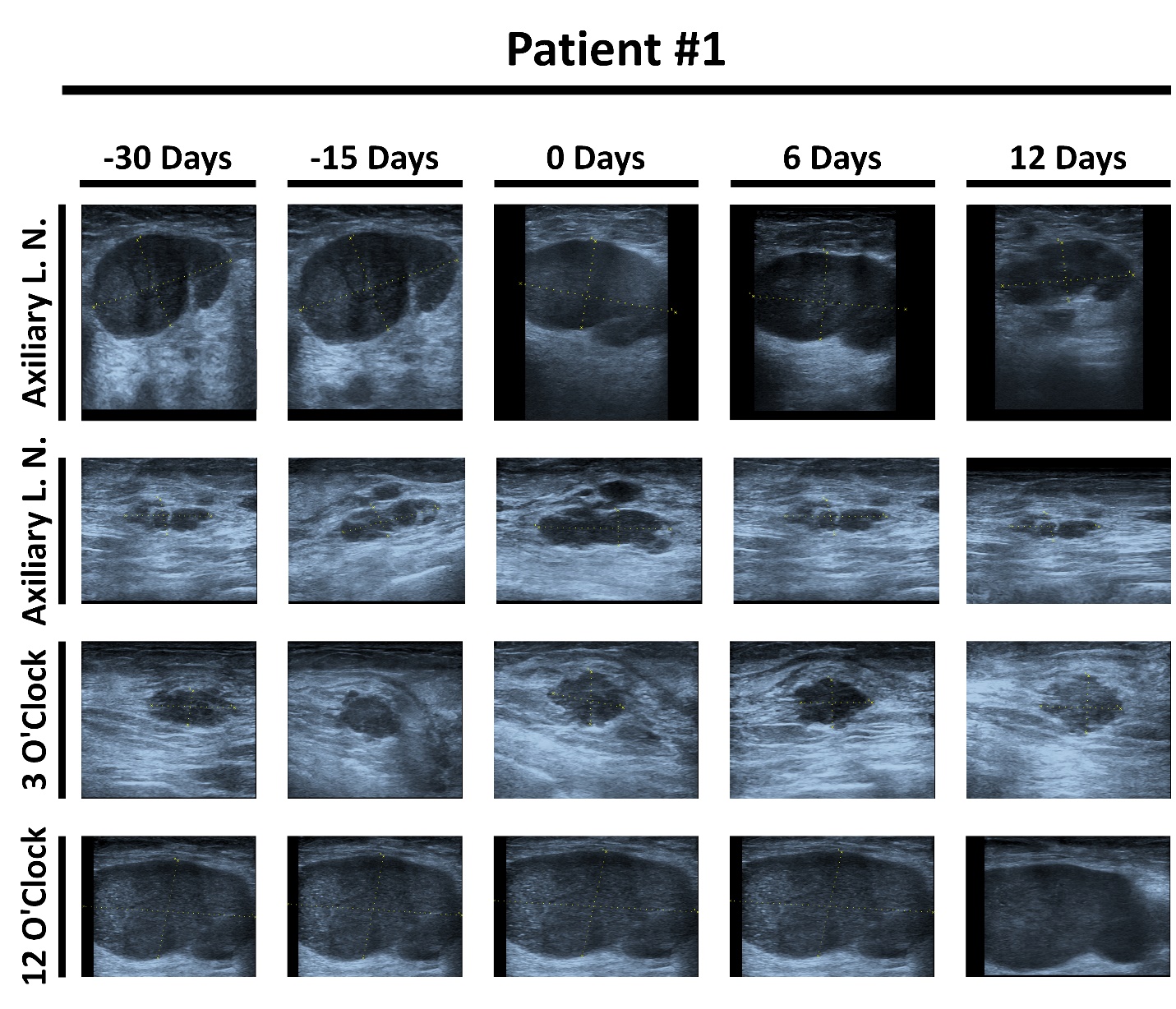


**Figure S4**, Serial sonography images of the breast and axillary masses in patient ID#1, 30 and 15 days before the PECT, followed by days 0, 6, and 12 of the positive electrostatic charge treating.

***
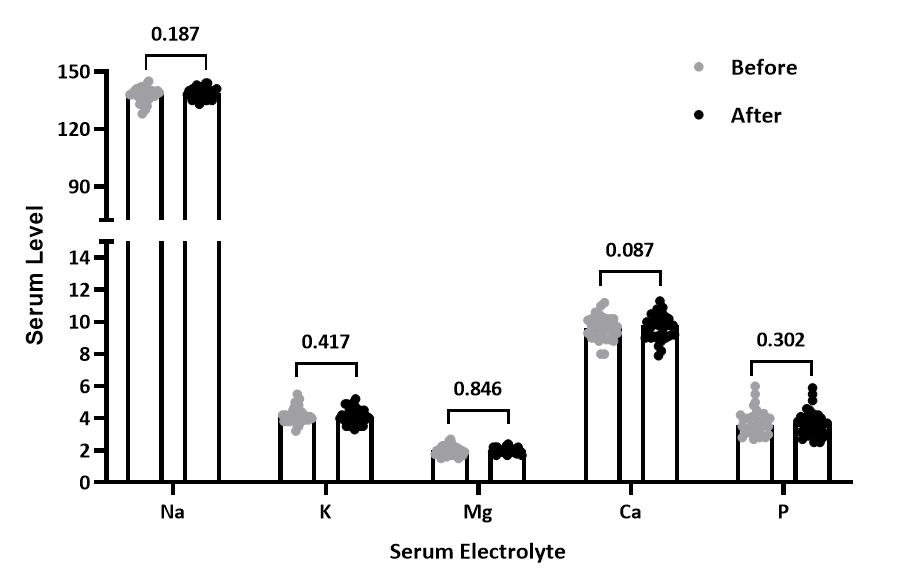
***

**Figure S5,** Serum electrolytes values of all 36 patients before and after PECT. The graph shows that PECT has not induced any significant changes in the serum electrolytes of the patients (Paired T-Test, P-value> 0.05)

Caspase-3, or cysteine-aspartic acid protease 3, is an enzyme involved in programmed cell death, also known as apoptosis. Caspase-3, a pivotal mediator of programmed cell death or apoptosis, plays a crucial role in maintaining tissue homeostasis and controlling abnormal cell proliferation. Its activation is a hallmark of the apoptotic cascade, where it cleaves various cellular substrates, leading to cell dismantling and eventual death. In the context of cancer research and pathology, caspase-3 has emerged as a vital biomarker, offering insights into the extent of apoptosis within tumor tissues. Dysregulation of caspase-3 expression can signify disruptions in apoptosis, a phenomenon often associated with malignancy. Here, we present caspase-3 IHC expression of post-treatment biopsies of two patients, patients #1 and #13.

High caspase-3 IHC expression typically indicates an increased level of apoptotic activity within the tissue or cells being analyzed. This can be indicative of various biological processes such as tissue remodeling, normal development, or response to treatment. Conversely, low caspase-3 IHC expression suggests reduced apoptotic activity.

After a comprehensive evaluation of the entire slide section, two pathologists provided us with their scores, which were based on their mutual diagnosis. We have included an image of each patient's caspase-3 IHC expression in Figure S6 to demonstrate the level of expression in each sample. As it is evident, patient #1 caspase-3 IHC expression scores 3 in terms of intensity which indicates strong staining, and score 1 in terms of proportion scores (<10% of tumor with strong intensity-SI). The strong expression intensity of the post-treatment biopsy of patient #1 clearly depicts an increased level of apoptotic activity, which definitely pertains to the applied PECT.

Patient #13 caspase-3 IHC expression result scores 3 in terms of intensity which indicates moderate staining and scores 1 in terms of proportion scores (<10% of tumor with strong intensity-SI). Results clearly indicate the effectiveness of the PECT. Scores indicate each level of expression and proportion as follows:

| **Intensity score** | **Proportion score** |
| --- | --- |
| - no or weak staining: Score=1 - moderate staining: Score=2 - Strong staining: Score=3 | - <10% of tumors with strong intensity (SI): Score=1 - = 10% ≤ strong intensity < 1/3 of tumor”: Score=2 - 1/3 ≤ strong intensity < 2/3 of tumor” Score=3 - strong intensity ≥ 2/3 of tumor” Score=4 |


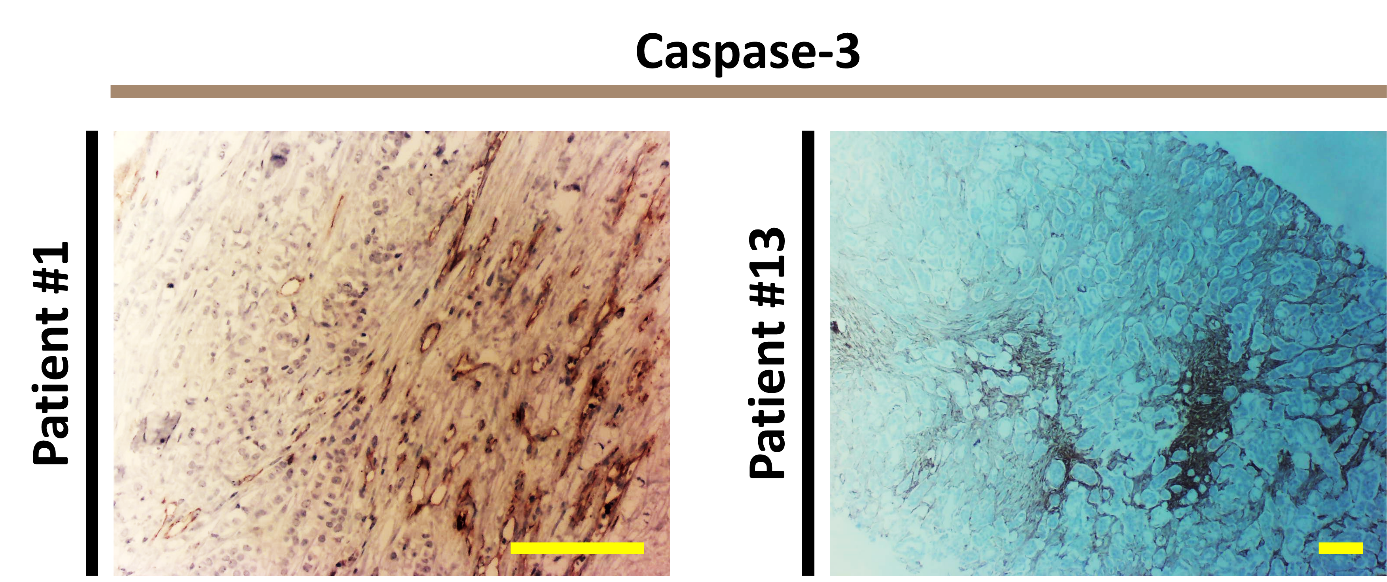


**Figure S6,** Post-treatment caspase-3 immunohistochemical (IHC) of the patients #1 and #13. Scale bars are set to 20 µm.

**Movie S1,** Interview with patient ID#7, a 64-year-old man with papillary thyroid carcinoma, who got three courses of PECT, resulted in reduced size of the posterior auricular (PA), left para-pharyngeal tumor (LPPT), and left retro-pharyngeal lymph node (LRPLN) tumors, with degradation of metastatic lung nodules, and reduced level of antithyroglobulin marker

**Movie S2,** Interview with patient ID#9, a 54-year-old woman with metastatic invasive ductal carcinoma of the breast, who got PECT, led to extensive healing of the cancer-induced ulcer, drastic reduction in tumor-induced edema, and reduced levels of CA15-3 markers.
